# Management of Atopic Dermatitis Via Oral and Topical Administration of Herbs in Murine Model: A Systematic Review

**DOI:** 10.3389/fphar.2022.785782

**Published:** 2022-05-24

**Authors:** Vivi Nur Khalieda Mohd Kasim, Siti Mahirah Noble, Kong Yen Liew, Ji Wei Tan, Daud Ahmad Israf, Chau Ling Tham

**Affiliations:** ^1^ Department of Biomedical Science, Faculty of Medicine and Health Sciences, Universiti Putra Malaysia, Serdang, Malaysia; ^2^ School of Science, Monash University Malaysia, Subang Jaya, Malaysia

**Keywords:** herbs, atopic dermatitis, topical, oral, murine model, IgE, Th2 cytokines, systematic review

## Abstract

Over the past few decades, complementary and alternative medicine (CAM) using herbs, or their active constituents have garnered substantial attention in the management of a chronic and relapsing inflammatory skin disorder called atopic dermatitis (AD), particularly in attenuating disease recurrence and maintaining long-term remission. In Eastern Asian countries including China, Korea and Taiwan, herbal medicine available in both topical and oral preparation plays a significant role in treating skin diseases like AD as they possibly confer high anti-inflammatory properties and immunomodulatory functions. Conventional murine models of AD have been employed in drug discovery to provide scientific evidence for conclusive and specific pharmacological effects elicited by the use of traditional herbs and their active constituents. Coupled with the goal to develop safe and effective novel therapeutic agents for AD, this systematic review consists of a summary of 103 articles on both orally and topically administered herbs and their active constituents in the murine model, whereby articles were screened and selected *via* a specialized framework known as PICO (Population, Intervention, Comparator and Outcome). The objectives of this review paper were to identify the efficacy of oral and topical administered herbs along with their active constituents in alleviating AD and the underlying mechanism of actions, as well as the animal models and choice of inducer agents used in these studies. The main outcome on the efficacy of the majority of the herbs and their active constituents illustrated suppression of Th2 response as well as improvements in the severity of AD lesions, suppression of Immunoglobulin E (IgE) concentration and mast cell infiltration. The majority of these studies used BALB/c mice followed by NC/Nga mice (commonly used gender–male; commonly used age group – 6–8 weeks). The most used agent in inducing AD was 2, 4-Dinitrochlorobenzene (DNCB), and the average induction period for both oral and topical administered herbs and their active constituents in AD experiments lasted between 3 and 4 weeks. In light of these findings, this review paper could potentially assist researchers in exploring the potential candidate herbs and their active constituents using murine model for the amelioration of AD.

## Introduction

Atopic Dermatitis (AD) is a chronic skin inflammatory disorder affecting 20% of children and up to 10% of adults worldwide (Laughter et al., 2021). Clinical manifestations of AD include dryness, erythema, itchy skin, and histological findings illustrate spongiosis and infiltration of inflammatory cells around the upper dermal layer (Han et al., 2016). Immune dysregulation as well as a plethora of genetic and environmental factors significantly contribute to the multifactorial etiology and complexed pathophysiology of a debilitating skin disorder like AD. With regards to immune dysregulation, AD has long been understood to predominantly express skewness towards a systemic T-helper type 2 (Th2)-dominant immune response (Renert-Yuval et al., 2021). With the expression of a Th2-skewed immunity, AD patients tend to exhibit an increase in cytokine expression, with interleukins 4 and 13 (IL-4 and IL-13) being the two pivotal cytokines that orchestrate the pathogenesis of this disease as well as elevated levels of high-affinity IgE receptor known as Fc epsilon receptor I (FcεRI). Studies have reported that the initiation of AD is closely associated with Th2 cytokines whereas the disease progression and chronicity are correlated with a delayed surge of Th1 cytokines mainly interferon- γ (IFN- γ) in the chronic phase, thus presenting itself as a biphasic inflammation (Grewe et al., 1998). Amongst the many prevalent hallmarks of AD, it is often correlated with a high level of circulating IgE and an elevated mast cells (MC) count in AD skin lesions (An et al., 2020).

Originally deriving from the bone marrow, MCs are the only terminally differentiated hematopoietic cells that express the c-Kit tyrosine kinase receptor and its ligand stem cell factor (SCF), to synergistically aid in mast cell proliferation and maturation in peripheral tissues (Mekori et al., 1995). Extensive studies in the literature have established the role of MCs as key contributors in the development and propagation of IgE-mediated hypersensitivity reactions as well as in innate and adaptive immune responses. Additionally, MCs are eminently located around the blood and lymphatic vessels, hair follicles and glandular structures of the skin. Mast cells found in the skin could be easily distinguished from mucosal MCs by their protease content as the former contains both chymase and tryptase in their granules whereas only tryptase is present in the latter (Stone et al., 2010). Upon exposure and sensitization to an allergen, activated B cells undergo differentiation into plasma cells to produce IgE, which readily cross-links with an antigen *via* high-affinity IgE receptors (FcεRI) on the MCs surface. This in turn stimulates IgE-mediated activation of MCs whereby subsequent degranulation induces the release of cytokines (i.e., IL-4, IL-5 and IL-13), preformed lipid mediators namely leukotrienes and platelet activating factor (PAF) which could further intensify the progression of AD (Kulka and Befus, 2003).

To date, the management of AD mainly focuses on the restoration of a normal functional skin barrier as well as to elicit anti-inflammatory and immunosuppressing effects, in a stepwise manner according to disease severity and symptoms (Lebwohl et al., 2013). Currently, standard treatment regimens for AD involve the use of moisturizing emollients, topical corticosteroids (TCs) and topical calcineurin inhibitors (TCIs) for mild-to-moderate AD; and the reliance on systemic immunosuppressant drugs such as azathioprine and mycophenolate mofetil or phototherapy, especially for more severe AD cases (Akhavan and Rudikoff, 2008; Lee HJ et al., 2016). However, studies have emphasized an increased risk of unwanted side effects such as skin atrophy, telangiectasia, acneiform eruptions and many more due to long-term usage of TCs and TCIs. Consequently, to address an unmet need in establishing a “gold standard” treatment for AD, profound interest lies in unravelling the development of a safe, efficacious as well as cost-effective anti-atopic agents derived from traditional herbs or natural sources (Choi JH et al., 2017; Nygaard et al., 2017).

Recent studies are actively demonstrating the potential pharmacological actions of traditional herbs and their active constituents as they might possess anti-inflammatory, antibacterial, antifungal, immunosuppressive activities and many more. *Aloe vera* (L.) Burm.f. is a plant widely prized in the medicinal, skincare and beauty industry owing to its wound healing and immunomodulatory properties. Based on a review carried out by Sánchez et al. (2020), the Chinese, Ayurvedic and Arabian medicine highly utilize *Aloe vera* (L.) Burm.f. as a treatment for constipation, helminth infections, and eczema respectively. In studies performed by Kim et al. (2010) and Finberg et al. (2015), the efficacy of a medicinal plant like *Aloe vera* (L.) Burm.f. in treating AD could be proven by its ability to modulate immunological responses in AD when administered *via* oral and topical routes. Once known as folk medicine, this stemless plant has gained a well-deserved reputation in ameliorating AD due to its profound anti-inflammatory properties and effectiveness in wound healing (Zari and Zari, 2015). Moreover, local inhabitants have been using herbs as one of the methods in managing diseases including skin related problems. As evidently seen in Nigeria, residents in Keffi are known to utilize the leaves of *Senna alata* (L.) Roxb. as a treatment for eczema by applying the herb extract to the affected areas (Mowobi et al., 2016) and this herb was proven to elicit dermatophytic activities due to its bioactive compounds (Oladeji et al., 2020). As reported by Hennebelle et al. (2009), *Senna alata* (L.) Roxb. was used not only as a topical treatment but also as an oral medicament against skin diseases including eczema. In consolidation, *Senna alata* (L.) Roxb. and *Aloe vera* (L.) Burm.f. were administered *via* the two most common routes of drugs administration, which further elucidates the primary focus of this review on highlighting both oral and topical routes of administration. A systematic review can therefore assist in analyzing the recent and relevant data from studies investigating the potential therapeutic effects of herbs and their active constituents on AD *via* two common routes of drug administration namely oral and topical. On top of that, an astonishing array of animal models have been developed in the field of skin diseases for drug discovery particularly in mice whereby they develop spontaneous skin lesions after prolonged exposure to various allergens and closely mimic the features of human AD. Therefore, this systematic review consists of a total of 103 *in vivo* studies of AD models that were being divided into two categories with the first section studying the oral administration of herbs and their active constituents (*n* = 48) followed by the topical route (*n* = 55). This review paper was commissioned:

i. to screen recent 6 years of publications (2014–2020) on oral and topical administration of herbs and their active constituents in murine models of AD
ii. to refine the suitable conditions for animal experimentation on AD by identifying the choice of animal models and inducer agents used
iii. to elucidate the presumed underlying mechanism of actions of oral and topical administered herbs and their active constituents as well as their efficacies

## Methods

### Search Strategy

**FIGURE 1:**
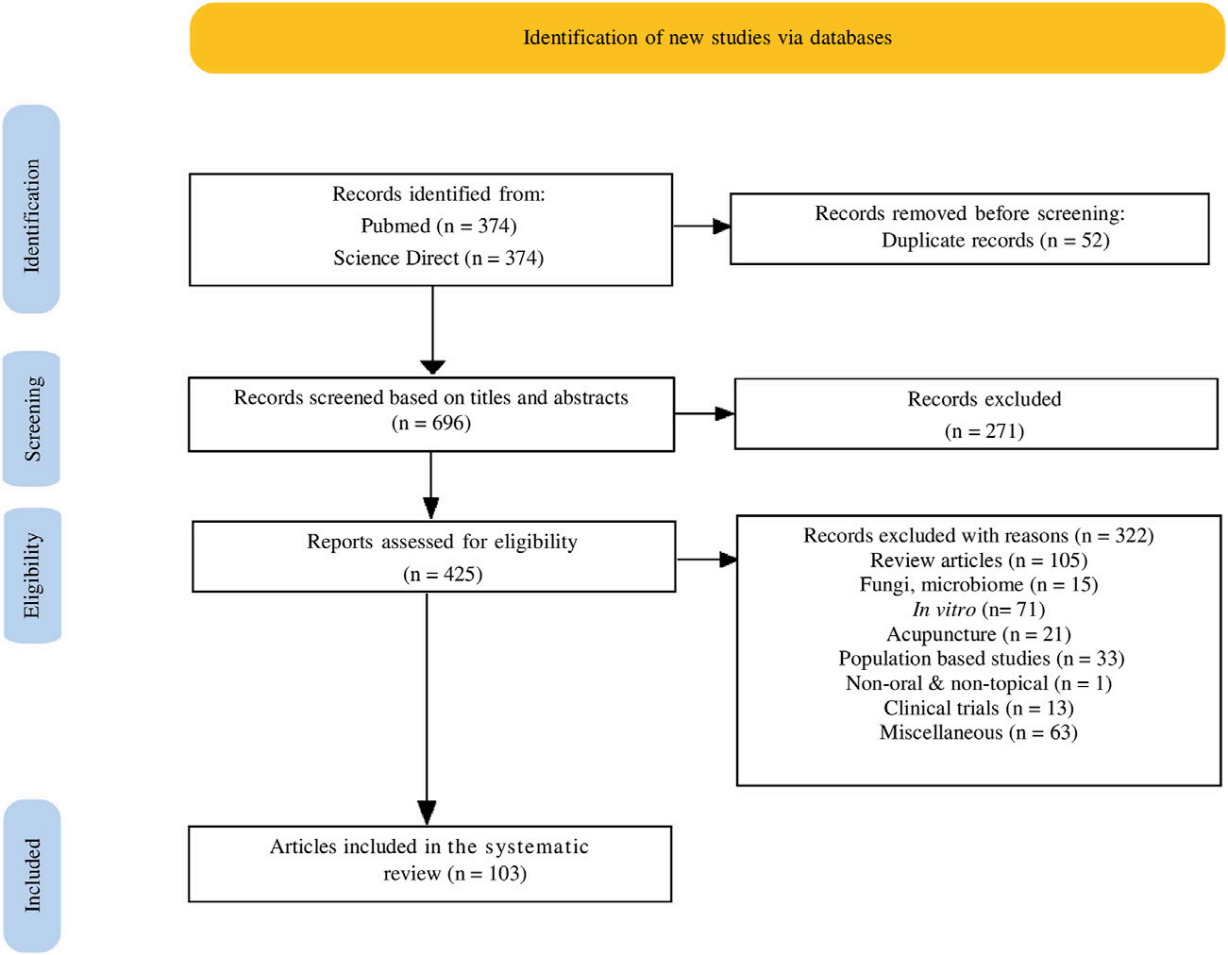
Flow chart illustrating the search strategy and study selection process from Pubmed and Science Direct according to the PRISMA statement.

Relevant articles in the literature were identified through systematic searches in two electronic databases, namely PubMed and ScienceDirect. The search terms comprised of two components: *1*) intervention or exposure to herb and *2*) disease of interest, “atopic dermatitis” or “eczema.” The Preferred Reporting Items for Systematic Reviews and Meta-analysis (PRISMA) criteria were followed in this study (Shamseer et al., 2015). PubMed thesaurus and MeSH term were used and filter was activated to select full text and publication year from 2014 to 2020. An additional filter on species “Other Animal” was also selected to focus on animal studies. ScienceDirect search was generated by truncating a term with an asterisk (*) as well as with the use of Boolean operators such as “AND” and “OR” to narrow the search to more manageable and relevant articles. After the initial search, refine filter was used to narrow down the articles to those published between 2014 and 2020. Owing to the absence of an animal exclusion search filter on ScienceDirect, manual searching was done to identify all relevant animal studies. The detailed search strategies for the two databases are presented in Table 1, where as the process and result of the systematic search are displayed in Figure 1. The flow diagram was created using an online tool that conforms to the PRISMA2020 Statement (Haddaway et al., 2021).

**TABLE 1:** Respective search strategies applied for PubMed and Science Direct.

	PubMed	Science direct
Intervention	(Herb* OR “herbal medicine” OR “medicinal plant” OR “Traditional Chinese Medicine” OR “Korean medicine” OR kampo OR Ayurveda OR ethnobotany)	(Herb* OR “herbal medicine” OR “medicinal plant” OR “Traditional Chinese Medicine” OR “Korean medicine” OR kampo OR Ayurveda OR ethnobotany)
Disease of interest	Atopic dermatitis OR eczema	Atopic dermatitis OR eczema
Outcome measures	Not included in the search term	Not included in the search term
Filter	2014–2020 Full-text available Other animals	2014–2020 Research articles

### Eligibility Criteria

The inclusion criteria are *1*) herbs (extract, mixture and active constituents from the herbs), *2*) AD or eczema in murine model. In contrast, the exclusion criteria are *1*) review articles, *2*) fungi, microbiome, *3*) *in vitro* studies, *4*) acupuncture, *5*) population-based studies, *6*) non-oral and non-topical route of herb administrations, *7*) clinical trials, *8*) miscellaneous articles (food chemistry, non-english, *etc*.) The eligibility criteria according to the PICO approach (population, intervention, comparator and, outcome) are as follows:

• Population: murine model with no restriction on gender, age and weight.
• Interventions: herbal extracts, complex mixtures of herbs or isolated active constituents from distinct parts of herbs.
• Comparator: a group that received control drugs (corticosteroids, calcineurin inhibitors or others).
• Outcomes: severity of AD lesions, serum IgE levels, infiltration of mast cells, Th1 and Th2 cytokine concentrations and other relevant parameters associated with AD.
• Study design: murine experimental research.

### Study Appraisal and Selection

Study selection was conducted independently by two reviewers (VK and SN) who searched the articles in PubMed and ScienceDirect databases. Irrelevant duplicate articles were crosschecked and removed by both authors and the remaining articles were screened based on the titles and abstracts. The relevant articles were further assessed to ensure that they comply with the eligibility criteria. Any contradictions between the authors were settled by consensus. Excluded articles were recorded with reasons. Subsequently, eligible literature was analyzed by the authors to extract the relevant information.

### Data Extraction

Data from all articles that met the criteria were extracted. Basic characteristics of the animals (age, gender and species) and experimental procedures including the inducers, the usage of barrier disruption tools, investigation sites and the control drugs were tabulated. Experimental outcomes and proposed mechanisms were listed systematically. This was followed by the preparations and chemical analysis of the herbs and their constituents.

### Risk of Bias

**FIGURE 2:**
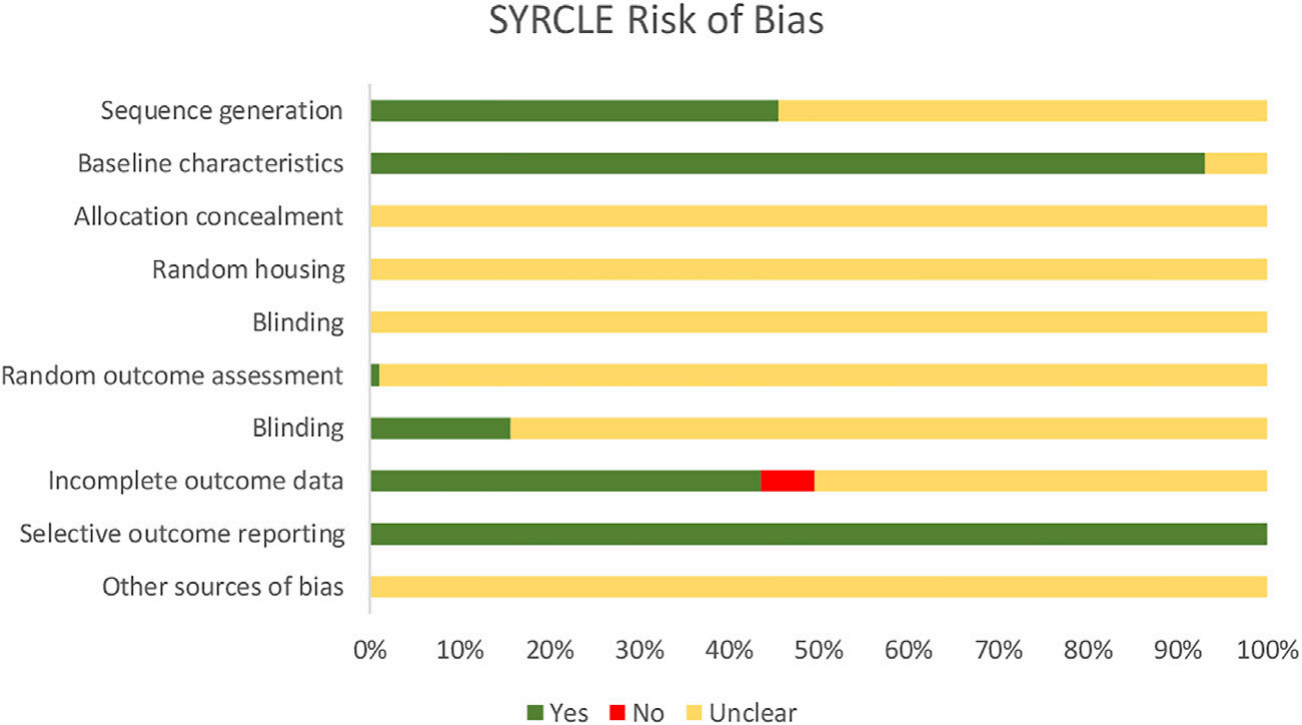
Assessment of the risk of bias (SYRCLE) for selected studies, presented as the percentages of the total.

Risk of Bias (RoB) assessment was performed using the SYstematic Review Centre for Laboratory Animal Experimentation (SYRCLE). The types of biases under RoB assessment include selection bias (sequence generation, baseline characteristics, allocation concealment), performance bias (random housing, blinding), detection bias (random outcome assessment, blinding), attrition bias (incomplete outcome data), reporting bias (selective outcome reporting) and other sources of bias. For each bias, there are different domains which constitute the ten entries for RoB tool for animal research. Each study was screened for all the entries and jotted with “yes” for a low risk of bias, “no” for a high risk of bias and “unclear” for an unclear risk of bias as presented in Figure 2.

## Results

### Risk of Bias

Risk of Bias (RoB) tool for animal intervention research in this sysmatic review is presented by SYRCLE (Hooijmans et al., 2014). SYRCLE is a tool for assessing the risk of bias in animal studies which consists of six types of bias. Under the selection bias, there are three domains. First is sequence generation. Almost half of the studies (45.54%) reported on the sequence generation that refers to the random sequence of the animal especially on the grouping. Second is baseline characteristics. Most studies (93.07%) mentioned the age, gender and weight of the animal in the methodology which proved that the experiments were started with animals having similar characteristics before the induction of AD. Third is allocation concealment. However, none of the studies mentioned that the information on the allocation of the animals were concealed from the investigators. Next, performance bias that consists of random housing and blinding were not reported in any studies. Under detection bias there are random outcome assessment and blinding. Only one study by Sur et al. (2017) reported random outcome assessment where the investigators randomly picked the animals before proceeding with the assessment. In only 15.84% of the studies, the outcome assessors were reported to be blinded. Incomplete outcome data under attrition bias revealed that 43.56% of the studies included all animals in the analysis. Reporting bias refers to whether or not the published report included all expected outcomes. All 103 studies reported expected oucomes when comparing methods and results section which defined as low risk of reporting bias. Other sources of bias such as the inappropriate influence of funders and the addition of new animals to replace dropout data were not reported in any of the studies. Nevertheless, these findings were expected as a survey disclosed that methodology and materials are poorly reported for experiments using animals, and the users of SYRCLE also mentioned that they had to judge a large number of entries as “unclear” (Kilkenny et al., 2009; Hooijmans et al., 2014).

### Oral Administration of Herbs and Their Active Constituents

#### Basic Characteristics of the Included Studies

In this review, 48 studies on orally administered herbs and their active constituents were analyzed with majority (*n* = 32) of them being based in Korea, 13 in China and the remaining three articles were from Japan, Taiwan and Pakistan, respectively. All the articles included in this review were rigorously assessed and screened according to a specialized search strategy known as PICO. The choice of intervention used during the screening process was “herb” which was being divided into three categories based on their formulation namely, extract, mixture, and compound. Most of the studies worked on the extracts (*n* = 20), followed by herb mixtures (*n* = 19) and compounds isolated from herbs (*n* = 8) as shown in Supplementary Table S1. The preparation steps of the herbs and their active constituents were shown in Supplementary Table S2. In addition, only one study focused on oil extracted from herb. None of the studies in this review was reported to work on the same herb using same route of administration. Two studies conducted by Tsang et al. (2016) and Aslam et al*.* (2018) were identified to work on a mixture of herbs named Pentaherbs and compound named Thymoquinone, respectively, *via* both oral and topical routes of administration.

#### Drug Control

The efficacy and safety of these novel oral herbal agents in the treatment of AD was corroborated with the use of well-established treatment modalities against AD. The choice of positive controls or comparison drugs in these studies varied from dexamethasone (*n* = 18), prednisolone (*n* = 5) and four individual studies by Ok et al. (2018) used terfenadine, Wu et al. (2014) used quercetin, Chen et al. (2017) used cetirizine and Aslam et al*.* (2018) used tacrolimus. However, 20 studies did not include a positive control drug. Lastly, a study by Zheng et al. (2019) was identified to use a total of three positive control drugs namely dexamethasone, loratadine and montelukast.

#### Animal Models Used

Murine models were the choice of population (P) for all the studies included in this review, to investigate the anti-AD effects of both oral and topical herbal administrations in an *in vivo* setting. Out of the 48 studies involving the oral route of herbal administration, male murine models (*n* = 28) and female murine models (*n* = 14) were used, however sex of the mice was not mentioned in six of the remaining studies. Moreover, the animal species also differed as 47 studies were identified to use mice of various strains; with BALB/C mice (*n* = 31) being the most popular strain, followed by NC/Nga mice (*n* = 14), C57BL/6J mice (*n* = 1), IL-4/Luc/CNS-1 transgenic mice (*n* = 1) and Sprague Dawley rats (*n* = 1). With reference to Table 2, induction of AD-like skin lesions was mostly achieved by the chemical exposure to 2, 4-Dinitrochlorobenzene (DNCB) as stated in 20 studies followed by 2, 4-Dinitrofluorobenzene (DNFB) as mentioned in eight studies. Six studies used *Dermatophagoides farinae* (DfE) and less than five studies used each of these inducers; fluorescein isothiocyanate (FITC), ovalbumin, oxazolone, phthalic anhydride (PA) and trimellitic anhydride (TMA). Interestingly, five studies were investigated to use a combination of inducers namely acetone with DNFB, 2, 4, 6-trinitro-1-chrolobenzene (TNCB) with ovalbumin and lastly, DfE with DNCB. In addition, mice in six studies received treatment with sodium dodecyl sulfate (SDS) to facilitate skin barrier perturbation and surgical tape stripping was performed in seven studies. Most of the studies administered the inducer on areas which include dorsal skin, ear or a combination of both as indicated in Table 2.

**TABLE 2:** Basic characteristics of the murine models and experimental procedures from studies involving oral treatments of herbs and their active constituents.

Age/Sex/Model species	Induction of AD-like skin lesions	Barrier disruption	Positive control drug	Investigation site	References
4w/M/BALB/c mouse	DNCB	N/A	1 mg/kg Dexamethasone	Dorsal skin	Lee JH et al. (2019)
5w/M/BALB/c mouse	DNCB	N/A	10 mg/kg Terfenadine	Dorsal skin	Ok et al. (2018)
5w/M/BALB/c mouse	DNCB	N/A	1 mg/kg Dexamethasone	Dorsal skin and ear	Yang et al. (2018)
5w/M/BALB/c mouse	DNCB	N/A	1 mg/kg Dexamethasone	Dorsal skin and ear	Zari and Zari, (2015)
5w/M/BALB/c mouse	DNCB	N/A	N/A	Dorsal skin	Jung et al. (2014)
5w/M/BALB/c mouse	DNCB	N/A	N/A	Dorsal skin	Park JH et al. (2019)
5w/M/BALB/c mouse	DNFB	N/A	N/A	Dorsal skin	Kee et al. (2017)
5w/M/BALB/c mouse	DNFB	N/A	N/A	Dorsal skin	Jeon et al. (2015)
6w/M/BALB/c mouse	DNCB	N/A	N/A	Dorsal skin	Ku et al. (2017)
6w/M/BALB/c mouse	DNCB	N/A	N/A	Dorsal skin	Kim SR et al. (2014)
6–7w/M/BALB/c mouse	DfE	N/A	N/A	Ear	Makino et al. (2014)
12w/M/BALB/c mouse	TMA	N/A	N/A	Ear	Sur et al. (2017)
M/BALB/c mouse	DNCB	N/A	30 mg/kg Prednisolone	Dorsal skin and ear	Song-lin et al. (2014)
4w/F/BALB/c mouse	DNCB and HDM (house dust mite)	Tape	Dexamethasone (dose not indicated)	Ear	Bae et al. (2016)
5w/F/BALB/c mouse	Ovalbumin	Tape	1 mg/kg Dexamethasone	Dorsal skin	Kong et al. (2015)
6w/F/BALB/c mouse	DNCB and DfE	Tape	N/A	Ear	Choi et al. (2014b)
6w/F/BALB/c mouse	DNCB and DfE	Tape	1 mg/kg Dexamethasone	Ear	Jeong et al. (2018)
6w/F/BALB/c mouse	Ovalbumin	Tape	1.3 mg/kg Cetirizine	Dorsal skin	Chen et al. (2017)
6–8w/F/BALB/c mouse	DNCB	N/A	10 mg/kg Prednisolone	Dorsal skin	Meng et al. (2019)
6–8w/F/BALB/c mouse	DNCB	N/A	2.5 mg/kg Dexamethasone	Dorsal skin and ear	Chen et al. (2020)
6–10w/BALB/c mouse	FITC	N/A	N/A	Ear	Tao et al. (2017)
6–10w/BALB/c mouse	FITC	N/A	0.67 mg/kg Dexamethasone	Ear	Wang et al. (2017)
8w/F/BALB/c mouse	DNCB	N/A	N/A	Dorsal skin and ear	Han et al. (2016)
8w/F/BALB/c mouse	Acetone and DNFB	N/A	0.039 mg/kg Dexamethasone	Abdominal and dorsal skin	Han et al. (2018)
8w/F/BALB/c mouse	TNCB and ovalbumin	N/A	1.6 mg/kg Quercetin	Dorsal skin	Wu et al. (2014)
8w/F/BALB/c mouse	DNFB	N/A	10 nM Dexamethasone	Dorsal skin	Moon et al. (2019)
8w/F/BALB/c mouse	Oxazolone	N/A	2.5 mg/ml Dexamethasone	Dorsal skin and ear	Tsang et al. (2016)
F/BALB/c mouse	DNCB	N/A	30 mg/kg Tacrolimus	Ear	Aslam et al. (2018)
5w/BALB/c mouse	DNCB	N/A	N/A	Abdominal and dorsal skin	Zhanxue et al. (2018)
6–8w/BALB/c mouse	FITC	N/A	0.67 mg/kg Dexamethasone	Abdominal skin and ear	Xiao-Tong et al. (2019)
BALB/c mouse	FITC	N/A	0.67 mg/kg Dexamethasone	Abdominal skin and ear	Zheng et al. (2019)
			1.3 mg/kg Loratadine		
			1.3 mg/kg Montelukast		
3w/M/NC/Nga mouse	DfE	5% SDS	N/A	Dorsal skin	Kim et al. (2020)
3w/M/NC/Nga mouse	DNFB	Tape and 5% SDS	N/A	Dorsal skin	Cha et al. (2016)
3w/M/NC/Nga mouse	DfE	Tape and 5% SDS	N/A	Dorsal skin	Cha HY et al. (2017)
5w/M/NC/Nga mouse	DNCB	N/A	3 mg/kg Prednisolone	Dorsal skin	Park et al. (2018)
6w/M/NC/Nga mouse	DNCB	N/A	3 mg/kg Dexamethasone	Dorsal skin	Park BK et al. (2014)
6w/M/NC/Nga mouse	DNCB	N/A	3 mg/kg Dexamethasone	Dorsal skin	Park et al. (2015)
6w/M/NC/Nga mouse	DNFB	N/A	N/A	Dorsal skin	Lee et al. (2017)
6w/M/NC/Nga mouse	DfE	4% SDS	5 mg/kg Dexamethasone	Dorsal skin	Kang et al. (2018)
6–8w/M/NC/Nga mouse	DNCB	N/A	3 mg/kg Dexamethasone	Dorsal skin, face and ear	Yan et al. (2019)
8w/M/NC/Nga mouse	DfE	4% SDS	3 mg/kg Prednisolone	Dorsal skin and ear	Lim et al. (2014b)
8w/M/NC/Nga mouse	DfE	4% SDS	3 mg/kg Prednisolone	Dorsal skin and ear	Lim et al. (2015)
M/NC/Nga mouse	DNFB	N/A	N/A	Abdominal and dorsal skin	Han et al. (2014a)
M/NC/Nga mouse	DNFB	N/A	0.039 mg/kg Dexamethasone	Abdominal and dorsal skin	Han et al. (2014b)
5w/F/NC/Nga mouse	DNCB	N/A	5 mg/kg Dexamethasone	Dorsal skin	Cha KJ et al. (2017)
4w/M/C57BL/6J mouse	DNFB	N/A	N/A	Dorsal skin	Park J et al. (2019)
8w/IL-4/Luc/CNS-1 transgenic mouse	PA	N/A	N/A	Ear	Kwak et al. (2017)
4w/M&F Lee/SD rat	DNCB	N/A	N/A	Dorsal skin and ear	Wan et al. (2016)

N/A, Not applicable.

### Topical Administration of Herbs

#### Basic Characteristics of the Included Studies

In this review, fifty-five studies were conducted across several East and South Asian countries with majority (*n* = 43) of them being based in Korea, seven in China, two in Japan, two in Taiwan and one study was performed in Pakistan. With cross-reference to Supplementary Table S3, the action of three different herbs namely, *Pyrus ussuriensis* Maxim., *Gardenia jasminoides* J.Ellis and *Stellera chamaejasme* L. were investigated repetitively in a few studies, whereas the subsequent 53 studies worked on diverse herbs of interest, in its extract (*n* = 32), mixture (*n* = 10) and compound (*n* = 11) forms. Interestingly, two studies in this review focused on studying the essential oil extracted from herbs. The preparation steps of the herbs and their active constituents were shown in Supplementary Table S4.

#### Drug Control

With close reference to Table 3, majority of the studies in this review used the two primary classes of drugs commonly used to treat AD as positive control drugs, which includes topical corticosteroids (*n* = 19) i.e., dexamethasone (*n* = 15), hydrocortisone (*n* = 2), mometasone (*n* = 1), betamethasone (*n* = 1) and topical calcineurin inhibitors (*n* = 16) i.e., tacrolimus (*n* = 14) and pimecrolimus (*n* = 2). However, 19 studies did not include a positive control drug and a study by Wu et al. (2020) confirmed the therapeutic actions of the herbal medicinal treatment against a pre-existing traditional Chinese medicine known as “Wu Dai ointment.”

**TABLE 3:** Basic characteristics of the murine models and experimental procedures from studies involving topical treatments of herbs and their active constituents.

Age/Sex/Model species	Induction of AD-like skin lesions		Barrier disruption	Positive control drug	Investigation site	References
5w/M/BALB/c mouse		DNCB	N/A	Betamethasone (% not indicated)	Dorsal skin (thoracic area)	Mechesso et al. (2019)
6w/M/BALB/c mouse		DNCB	N/A	N/A	Dorsal skin	Jung et al. (2015)
6w/M/BALB/c mouse		DNCB	N/A	Tacrolimus (% not indicated)	Dorsal skin	Hong et al. (2019)
6w/M/BALB/c mouse		DNCB	4% SDS	10 µM Dexamethasone	Dorsal skin	Choi et al. (2018)
6w/M/BALB/c mouse		DNCB	N/A	0.1% Tacrolimus	Dorsal skin	Ku et al. (2018)
8–10w/M/BALB/c mouse		DNFB	N/A	N/A	Both ears	Osada-Oka et al. (2018)
5w/F/BALB/c mouse		DNCB	N/A	10 µM Dexamethasone	Dorsal skin	Lee J et al. (2016)
6w/F/BALB/c mouse		DNCB and Oxazolone	N/A	N/A	Dorsal skin, ears	Lee et al. (2018)
6w/F/BALB/c mouse		DNCB	4% SDS	10 µM Dexamethasone	Dorsal skin	Choi et al. (2016)
6w/F/BALB/c mouse		DNCB	4% SDS	10 µM Dexamethasone	Dorsal skin	Choi YY et al. (2017b)
6w/F/BALB/c mouse		DNCB and DfE	Tape	N/A	Both ears	Choi and Kim, (2014a)
6w/F/BALB/c mouse		Oxazolone	N/A	N/A	Both ears	Jo et al. (2018)
6w/SKH-1 hairless mouse		DNCB	N/A	Pimecrolimus (1% Elidel)	Dorsal skin	
6w/F/BALB/c mouse		Oxazolone	N/A	N/A	Both ears	Jung et al. (2018)
6w/F/BALB/c mouse		Oxazolone	N/A	N/A	Both ears	Lee S et al. (2019)
6w/SKH-1 hairless mouse		DNCB	N/A	N/A	Dorsal skin	
6w/F/BALB/c mouse		DNCB	4% SDS	N/A	Dorsal skin	An et al. (2020)
6w/F/BALB/c mouse		DNCB	N/A	10 µM Dexamethasone	Dorsal skin	Choi YY et al. (2017a)
6w/F/BALB/c mouse		Oxazolone	N/A	N/A	Dorsal skin, ears	Jegal et al. (2017)
6w/F/SKH-1 hairless mouse		DNCB	N/A	N/A	Dorsal skin, ears	
7w/F/BALB/c mouse		DNCB	N/A	Wu Dai ointment	Dorsal skin	Wu et al. (2020)
7w/F/BALB/c mouse		DNCB	N/A	N/A	Dorsal skin	Lim et al. (2018)
7w/F/BALB/c mouse		DNCB	4% SDS	0.1 and 0.5% Dexamethasone	Dorsal skin	Lee HJ et al. (2016)
8w/F/BALB/c mouse		DNFB	N/A	5 mg/kg Dexamethasone	Dorsal skin	Ryu et al. (2018)
8w/F/BALB/c mouse		DNCB	N/A	N/A	Dorsal skin, both ears	Fang et al. (2015)
8w/F/BALB/c mouse		DNCB	N/A	N/A	Dorsal skin, ears	Huang et al. (2019)
8w/F/BALB/c mouse		Oxazolone	N/A	2.5 mg/ml Dexamethasone	Both ears	Tsang et al. (2016)
F/BALB/c mouse		DNCB	N/A	1% Tacrolimus	Both ears	Aslam et al. (2018)
4w/M/NC/Nga mouse		DfE	N/A	N/A	Dorsal skin, ears	Yang G et al. (2016)
6w/M/NC/Nga mouse		DNCB	N/A	N/A	Dorsal skin	Yang HJ et al. (2017)
6w/M/NC/Nga mouse		DNFB	N/A	N/A	Dorsal and abdominal skin	Han et al. (2014c)
6w/M/NC/Nga mouse		Biostir-AD	4% SDS	0.1% Tacrolimus	Dorsal skin	Ngo et al. (2020)
6w/M/NC/Nga mouse		DNCB	N/A	0.1% Dexamethasone	Dorsal skin	Yang IJ et al. (2016)
6w/M/NC/Nga mouse		DfE	4% SDS	0.1% Tacrolimus	Dorsal skin, ears	Sung and Kim, (2018)
7w/M/NC/Nga mouse		DNCB	N/A	3 mg/kg Dexamethasone	Dorsal skin	Kim et al. (2015)
8w/M/NC/Nga mouse		DfE	4% SDS	0.1% Tacrolimus	Dorsal neck, ears	Park et al. (2020)
8w/M/NC/Nga mouse		DfE	4% SDS	0.1% Tacrolimus	Dorsal skin, ears	Park SH et al. (2019)
8w/M/NC/Nga mouse		DfE	4% SDS	0.1% Tacrolimus	Dorsal skin, both ears	Sung et al. (2016)
8w/M/NC/Nga mouse		DfE	4% SDS	0.1% Tacrolimus	Dorsal skin, both ears	Sung et al. (2014)
8w/M/NC/Nga mouse		DfE	10% (w/v) SDS	0.1% Tacrolimus	Dorsal skin, both ears	Lee et al. (2014)
8w/M/NC/Nga mouse		DfE	4% SDS	0.1% Tacrolimus	Dorsal skin, both ears	Lim et al. (2014a)
8w/M/NC/Nga mouse		DfE	N/A	0.1% Tacrolimus	Upper dorsal skin, both ears	Ha et al. (2014)
8w/M/NC/Nga mouse		DfE	N/A	0.1% Tacrolimus	Upper dorsal skin, both ears	Ha et al. (2015)
4w/F/NC/Nga mouse		DNCB	N/A	0.1% Dexamethasone	Dorsal skin, ears	Cho et al. (2019)
4w/F/NC/Nga mouse		DNCB	N/A	0.1% Dexamethasone	Dorsal skin, ears	Cho et al. (2018)
4w/F/NC/Nga mouse		DNCB	N/A	0.1% Tacrolimus	Dorsal skin, face and both ears	Kim H et al. (2014)
4w/F/NC/Nga mouse		DNCB	N/A	N/A	Dorsal skin	Ham et al. (2019)
5w/F/NC/Nga mouse		DfE	N/A	0.1% Hydrocortisone	Dorsal skin	Park KH et al. (2014)
5w/F/SKH-1 hairless mouse		Oxazolone	N/A	0.1% Hydrocortisone	Dorsal skin	Kang et al. (2017)
6w/F/SKH-1 hairless mouse		DNCB	N/A	Pimecrolimus (1% Elidel)	Dorsal skin	Lee et al. (2020)
6w/F/SKH-1 hairless mouse		DNCB	N/A	N/A	Dorsal skin	Jo et al. (2018)
4w/M/Kunming mouse		DNCB	N/A	Dexamethasone and Paeonol cream (% not indicated)	Dorsal skin, right ear	Yang J et al. (2017)
8w/M/Kunming mouse		DNCB	N/A	Dexamethasone acetate (contain 0.075% dexamethasone)	Dorsal skin, right ear	Fan et al. (2019)
8w/M/Kunming mouse		DNCB	N/A	0.025% Dexamethasone	Dorsal skin, right ear	Fan et al. (2018)
5–8w/F/ICR mouse		DNCB	N/A	0.1% Mometasone furoate cream	Dorsal skin, right ear	Wang et al. (2018)
8w/HR-1 mouse		Phthalic anhydride	N/A	N/A	Dorsal skin, ears	Ho et al. (2018)
6–8w/F/C57BL/6 mouse		MC903 (vitamin D3 analogue)	N/A	N/A	Dorsal side of left ear	Hou et al. (2019)
8–10w/M/NC/Tnd mouse		N/A	N/A	N/A	Dorsal skin	Amagai et al. (2017)

N/A, Not applicable.

#### Animal Models Used

Murine models had been used in all the studies to investigate the anti-AD effects of novel topical herbal administration in an *in vivo* setting. Female mice were identified in 30 studies while male mice were used in 25 studies, however, a few studies did not disclose the gender of the mice. Categorically, the BALB/c mice model (*n* = 25) was most extensively used among the included studies, superseded by NC/Nga (*n* = 20), hairless mice (*n* = 6), Kunming mice (*n* = 3), ICR mice (*n* = 1), HR-1 mice (*n* = 1), C57BL/6 (*n* = 1) and NC/Tnd mice (*n* = 1). It is also noteworthy that three individual studies conducted by Jegal et al. (2017), Jo et al. (2018) and Lee JH et al. (2019), utilized two different mice species namely BALB/c and SKH-1 hairless mice. According to Erkes and Selvan (2014), variation in the type of mouse strains could lead to a substantial difference in the immune stimulatory ability of haptens used to induce AD, which in these three studies, were DNCB and oxazolone. In order to evoke AD-like skin lesions, repetitive cutaneous sensitization with house dust mite allergen namely *Dermatophagoides farinae* (DfE) and chemical induced irritation *i.e.,* DNCB, DNFB, oxazolone, MC903 (a synthetic derivative of vitamin D) and phthalic anhydride were used in 12 and 42 studies, respectively*.* However, a study by Amagai et al*.* (2017) on *Alpinia intermedia* Gagnep., differed substantively from the rest as they used NC/Tnd mice, a sub-strain of Nc/Nga developed from inbreeding to give spontaneous itch and eczematous skin which resembles AD. Hence, no induction agent was needed to elicit AD-like skin lesions in this particular mouse strain. Besides, sodium dodecyl sulfate (SDS) was applied to the AD lesions in 13 of the studies for barrier disruption purposes, in which 12 received treatment with 4% (w/v) SDS, whereas only one study by Lee et al. (2014) applied 10% (w/v) SDS to mice skin. Mechanical skin injury by surgical tape stripping was only performed in a single study by Choi and Kim (2014a). On top of that, the majority of the studies in this review focused on investigating the dorsal skin, ears, or a combination of both as they were representative sites depicting the histological features of AD-like skin lesions.

## Discussion

Over the past few decades, there has been a tremendous surge in the public’s shifting interest towards the reliance on natural herbal remedies as alternative therapeutic options to conventional Western allopathic medicine to treat AD. Essentially, plant-based or herbal medicine are natural products consisting portions of plants or unpurified plant extracts that possess potential bioactive compounds offering a myriad of medicinal and nutritive values possibly working synergistically, to affect multiple targets that contribute to the pathogenesis of AD. To date, the cornerstone of AD management commonly involves therapeutic intervention with emollients, TCs, TCIs and systemic immunosuppressants, especially for more severe AD cases (Carr, 2013; Nygaard et al., 2017). Nonetheless, inter-individual drug response variability and the potential risk of adverse effects from long-term use such as skin atrophy, tachyphylaxis and telangiectasia, are the key rationale behind a shifting interest towards herbal drug development with lesser side effects (Thomsen 2014; Montes-Torres et al*.,* 2015). With that goal in mind, this systematic review was drawn from 103 studies evaluating the efficacy of novel herbs and their active constituents, synthesized from various preparations including crude extracts, compounds isolated from the herbs or mixtures of different herbs applied *via* oral and topical administration in *in vivo* murine AD models.

The use of murine models is needed to aid in drug discovery and testing of novel treatments as they are capable of mimicking different aspects of the pathophysiology of human AD. Mice models of AD are generally classified into three categories: *1*) mice induced by epicutaneous sensitization with allergens, *2*) spontaneous mice models and *3*) genetically modified mice that either over-express a transgene or lack of selective endogenous genes. In this review paper, the majority of the studies used allergen-induced AD models, which are relatively more convenient as the usage of exogenous allergens permits dose- and time-controlled induction (Kim et al., 2019). Also, out of 48 oral and 55 topical herbs administration studies involving AD in murine models, the majority used BALB/c mice strain (oral: *n* = 31, topical: *n* = 25), followed by NC/Nga (oral: *n* = 14, topical: *n* = 20). BALB/c mice have been widely known to trigger a Th2-dominant immune response, therefore, propagating the development of AD (Lee et al., 2010). On the other hand, NC/Nga mice were investigated to develop approximately 50% of spontaneous AD-like skin lesions when they are kept in conventional housing with uncontrolled air but remain normal and healthy under specific pathogen-free (SPF) conditions. However, this particular mouse model requires repeated applications of a hapten to fully develop AD (Shiohara et al., 2004).

Gender considerations remain an important facet in *in vivo* pharmacology studies as a substantial body of literature supports the notion that male mice are highly preferred in comparison to female due to the ease of handling, less variation in results and cost-effectiveness. Studies on orally administered herbs in this review correspond to this notion as male mice (*n* = 28) and female mice (*n* = 14) were used. Contrastingly, this was not constant with studies involving topical herb administration as female mice were found to lead the number with *n* = 30 and male (*n* = 25). However, this gap was not of significant difference and male mice are indeed more favourable in AD research. According to Wald and Wu (2010), female murine models confer a 4-day oestrous cycle which therefore accounts for the use of a larger number of animals as compared to males in order to keep their cycle in synchrony, and this in turn makes it less economical. Additionally, the fluctuation of hormones across their oestrus cycle results in behavioural variability in female mice as seen in the production of a female sex hormone called oestrogen which was reported to negatively regulate the wound healing process of AD-like skin lesions (Mukai et al., 2016). Taken together, researchers more typically eliminate the use of female murine models on the presupposition that they elicit intrinsic variation as compared to males, which could possibly interfere with the efficacy of herbs of interest in ameliorating AD. It was also brought to attention that a range between 6 and 8 weeks were the most commonly used murine age groups across the studies in this review, involving oral (*n* = 24) and topical (*n* = 43) herb administration. In murine, the maturation of T cells was at 8 weeks of age, which is crucial for the production of T-cell-dependent antibodies (Holladay and Smialowicz, 2000; Jackson et al., 2017).

A vast array of inducer agents was utilized across the studies on orally and topically administered herbs with the majority of them involving the use of DNCB (*n* = 52) either individually or in combination with DfE or Biostir-AD ointment (Choi and Kim, 2014a; Choi et al., 2014b; Bae et al., 2016; Jeong et al., 2018) and oxazolone (Jegal et al., 2017; Lee et al., 2018; Jo et al., 2019). DNCB exposure can create covalent conjugates with skin proteins to undergo further processing and presented to T cells for activation. Besides, repeated exposure to DNCB permits the accumulation of macrophages at the sensitization site, hence exemplifying an allergic inflammatory response as seen in AD (Yan et al., 2019). In fact, Ku et al. (2018) mentioned that morphological changes in the skin lesions of DNCB-sensitized mice revealed some of the major histopathological findings associated with human AD such as thickened epidermis, massive infiltration of CD4^+^ T-cells producing pro-inflammatory cytokines as well as increased infiltration of inflammatory cells i.e., mast cells and eosinophils. Apart from DNCB, several other chemical compounds were also used to induce AD-like lesions in topical application of herb section of this review, including DNFB (*n* = 3), oxazolone (*n* = 6), repetitive cutaneous sensitization with DfE ointment (*n* = 12), MC903 (*n* = 1), and phthalic anhydride (*n* = 1). As we dived into the induction period, it was noticeable that the average duration of exposure to an inducer lasted between 3 and 4 weeks, with the shortest being a week. However, in a study conducted by Jung et al. (2015), DNCB exposure lasted for approximately 8 weeks (∼55 days). DNFB (*n* = 8), DfE (*n* = 6) and FITC (*n* = 4) are among other inducers that were mentioned in this orally administered herb section of this review. In terms of duration, FITC marked the shortest period of AD induction with only 3 days then the mice were sacrificed. This is because the induction was only for the initial stage of AD and the articles from Tao et al. (2017) and Wang et al. (2017) did not mention any data on dermatitis score, serum IgE level and mast cell count which are the hallmarks of AD hence it was difficult to assess the AD severity. On the other hand, DfE recorded the longest period of AD induction which is 8 weeks.

Interestingly, nine out of 14 studies on topically administered herbs and their active constituents and five out of six studies on orally administered herbs and their active constituents were subjected to repeated application of DfE, subsequently after barrier disruption with a chemical substance mainly known as sodium dodecyl sulfate (SDS). SDS functions to facilitate the development of AD-like skin lesions by eliminating the lipid lamella of the stratum corneum. With reference to Supplementary Tables S1, S3, the oral and topical application of herbs and their active constituents was found to ameliorate AD symptoms *via* restoration of the skin barrier function (Cha et al., 2016; Yang JH et al., 2016; Cha HY et al., 2017; Cha KJ et al., 2017; Kang et al., 2018; Lee et al., 2018; Zhanxue et al., 2018; Cho et al., 2019; Jo et al., 2019; Park J et al., 2019; Park JH et al., 2019). Breakdown in the architecture of the stratum corneum in response to barrier disruption methods with either SDS or tape stripping, further permits the entry of allergens across the skin to interact with local antigen-presenting cells and immune effector cells. Previous research in the literature illustrated that repetitive application of DfE is capable of contributing to the onset and development of AD *via* the activation of inflammasomes in the epidermal keratinocytes (Jin et al., 2016). Restoration of an intact skin barrier in AD serves as a valuable therapeutic target since it acts as the first line of defence from various microbes and environmental allergens, as well as decreases in trans-epidermal water loss (TEWL). Subsequently, we found out that two studies of oral herbs and their active constituents' administration and 13 studies using topical herbs and their active constituents' administration were shown to decrease TEWL, whereas five studies on orally treated herbs and their active constituents and seven studies on topically treated herbs and their active constituents showed increased levels of epidermal barrier proteins such as filaggrin, involucrin and loricrin.

Until recently, the etiology of AD could possibly be accounted by two opposing hypotheses brought up by Silverberg and Silverberg (2015). The “outside-in” hypothesis supports the notion impairments in the epidermal barrier are solely responsible for the onset of inflammatory lesions in AD and an essential structural protein filaggrin, possesses a fundamental role in maintaining skin barrier integrity, skin hydration and pH by minimizing water loss (Li, 2014; Malik et al., 2017). In contrast, there are some who propose the “inside-out” hypothesis in which a dysregulated immune response is the key event that propagates the inflammatory cascade in AD and skin barrier disruption is an epiphenomenon (Boguniewicz and Leung, 2011; Silverberg and Silverberg, 2015). In this review, 102 studies could be accounted by the outside-in theory as murine models were introduced to the experimental allergens repeatedly in order to disrupt epidermal barrier integrity. Additionally, 12 studies in this review reported that oral and topical herbs and their active constituents' administration elicited an increase in levels of epidermal barrier proteins such as filaggrin, involucrin and loricrin. Hence, it allows us to justify that orally and topically administered herbs and their active constituents confers the potential to accelerate barrier recovery by up-regulating the expression of such vital structural proteins of the skin.

To fully comprehend the origins of AD, a molecular link has been well-documented between the elevated serum IgE levels and marked infiltration of mast cells. During AD inflammation, an allergen penetrates through the skin and gets readily bound to MCs by cross-linking to the high-affinity IgE receptor, FcɛRI, expressed on their cell surface (Brown et al*.,* 2008; Amin, 2012). Upon the activation of MCs, degranulation occurs within seconds, leading to the subsequent release of preformed secretory granules containing biologically active inflammatory mediators and proteins including histamine (cutaneous inductor of itch), PGs and leukotrienes, as well as serine proteases i.e. mast-cell chymase and tryptase (Kawakami et al., 2009; Siebenhaar et al., 2018). Among the 48 studies on orally administered herbs and their active constituents, 37 of them were reported to measure serum IgE levels and 22 studies measured the number of MCs and reported congruent decrease. Decreased levels in both of these crucial parameters suggest that the oral route of herbs and their active constituents’ administration is capable of alleviating the allergic responses. On the other hand, across the 45 studies investigating IgE levels as an outcome parameter for topically administered herbs and their active constituents, it was found that 39 of these studies measured serum IgE and six studies investigated plasma IgE levels. Fortunately, serum or blood IgE levels successfully decreased after treatment with topical herbs and their active constituents in all 45 studies. However, it is important to note that total serum IgE may not reflect accurate levels of systemic IgE since raised concentrations are present in many patients who have no evidence of allergy. As a result, accurate measures of allergen-specific IgE could assist in defining the bona fide mechanism of novel topically applied herbal drugs in immunity (Qiu et al., 2020). In addition, from the studies involving topical route of administration, 39 herbs and their active constituents were found to significantly decrease the extent of mast cell infiltration into the skin. Moreover, it seems that the reduction of MCs infiltration can be associated with a decrease in MCs degranulation as well as down-regulation of IL-4 and IL-13 cytokines secretion. Therefore, this implies that MCs may serve as a key contributing factor to the suppression of Th2 cytokines in AD-like skin lesions *via* topical herbs and their active constituents' administration.

The extensive array of mediators secreted from FcεRI-mediated mast cell degranulation can ultimately promote inflammation, resulting in the appearance of macroscopic and microscopic skin lesions which are associated with AD. The severity of AD-like skin lesions could be investigated macroscopically according to the acronym widely known as SCORAD (SCORing Atopic Dermatitis) index. This scoring system serves as a clinical tool to assess the extent and severity of eczema by evaluating the common characteristic skin symptoms of AD including *1*) erythema, *2*), erosion, *3*) dryness and *4*) lichenification. In our review, dermatitis severity was measured in 26 studies using oral administration of herbs in which all 26 herbs and their active constituents were able to reduce the dermatitis severity score. The same results were obtained for treatment with topical herbs and their active constituents as the dermatitis severity score was found to decrease in 19 studies. On top of that, there was a study performed by Aslam et al*.* (2018), investigating on a single compound named thymoquinone, which used both routes of drug administration. Results from this study illustrated that both routes were able to alleviate the dermatitis severity score in murine models of AD. However, more in-depth studies need to be performed to thoroughly compare the efficacy of thymoquinone *via* different routes of administration before definitive conclusions can be drawn.

The immunological hallmark of AD is characterized by a predominant expression of Th2-inflammatory cytokines (i.e., IL-4, IL-5 and IL-13) in the acute phase followed by a prolonged activation of Th1-type cytokines (i.e., IFN-γ and TNF-α) in the chronic phase of AD (Brunner et al., 2017; Choi et al., 2014b). As demonstrated in Supplementary Table S1, 19 studies showed a simultaneous decrease in both Th1 and Th2 cytokines after treatment with orally administered herbs and their active constituents. Apart from that, 11 articles were found to study the Th2 cytokines independently and results showed a reduction in the level of cytokines. Without much anticipation, four studies measured the cytokines released from both types of helper T cells reported an unpredictable finding as orally administered herbs and their active constituents decreased the level of Th2 cytokines but contrastingly, induced an increase in the level of Th1 cytokines (Wu et al., 2014; Kong et al., 2015; Wan et al., 2016; Han et al., 2018). A substantial body of literature have suggested that an aberrant Th2-immune response plays a key proximal role in the pathogenesis of AD, hence the two vital Th2 cytokines, IL-4 and IL-13 were investigated in 38 and 32 studies of topical administration of herb and their active constituents, respectively. Besides, another Th2-mediated cytokine, IL-5 was measured in 11 studies. In all the studies mentioned above, topical application of herbs and their active constituents resulted in a marked reduction of IL-4, IL-5 and IL-13 expression. To address the classical Th1/Th2 paradigm in AD, TNF-α and IFN-γ levels were investigated as representative Th1 cytokines in 24 and 16 studies, respectively. Across all these studies, topical herbal application induced a decrease in TNF-α and IFN-γ levels, however, this was not achieved in a study by Yang HJ et al. (2017) whereby an unexpected asynchronism in both Th1 cytokines was reported after treatment. This finding could be accounted by the “Th1/Th2 imbalance” in the immunological hallmark of AD, in which Th1 responses are capable of antagonizing the development of Th2 cells, hence producing a contrasting effect in the levels of both types of cytokines (Herberth et al., 2010). On the other hand, extremely low and undetected levels of IFN-γ were reported by Yang J et al. (2017), possibly due to a Th2-driven inflammation whereby IL-4 was able to truncate the key Th1 cytokines namely IFN-γ (Brunner et al., 2017).

Recent progress in molecular biology has provided insights into the potential role of nuclear factor kappa B (NF-κB) transcription factor family in the progression and maintenance of AD (Tanaka et al., 2007). Studies have illustrated that activation of the NF-κB pathway is accountable for the regulation and chronicity of inflammatory skin diseases like AD. NF-κB participates in the differentiation of T cells into various subsets comprising of Th1, Th2 and Th17. Moreover, NF-κB functions to increase the expression and transcription of numerous cytokines, growth factors and chemokines such as IL-1β, IL-6 and IL-8, which are highly linked to the inflammatory cascade in AD (Tak and Firestein, 2001; Liu et al., 2017). In this review, 10 articles on orally administered herbs and their active constituents managed to inhibit the NF-κB pathway and similar results were recorded from 10 articles on topically administered herbs and their active constituents. Apart from NF-κB pathway, the phosphorylation of mitogen-activated protein kinases (MAPKs) signalling pathway is closely associated with the production of pro-inflammatory mediators which could trigger and exacerbate AD. Owing to the importance of MAPK signalling molecules i.e. ERK, JNK and p38, in the activation, proliferation, migration and degranulation of immune cells involved in AD, therefore pharmacological inhibition of this pathway may serve as an attractive strategy for the treatment of allergic disorders (Huang et al., 2019). Inhibition of MAPK activation were seen in eight articles after administration of herbs and their active constituents *via* oral route whereas 11 studies illustrated an inhibition of MAPK phosphorylation after administration of herbs and their active constituents topically, as illustrated by a decrease in ERK, JNK and p38 protein kinases. As such, these findings propose that both NF-κB and MAPK signalling pathway serve as the potential avenues of investigative research in the development of a new therapeutic agent to treat AD.

**FIGURE 3:**
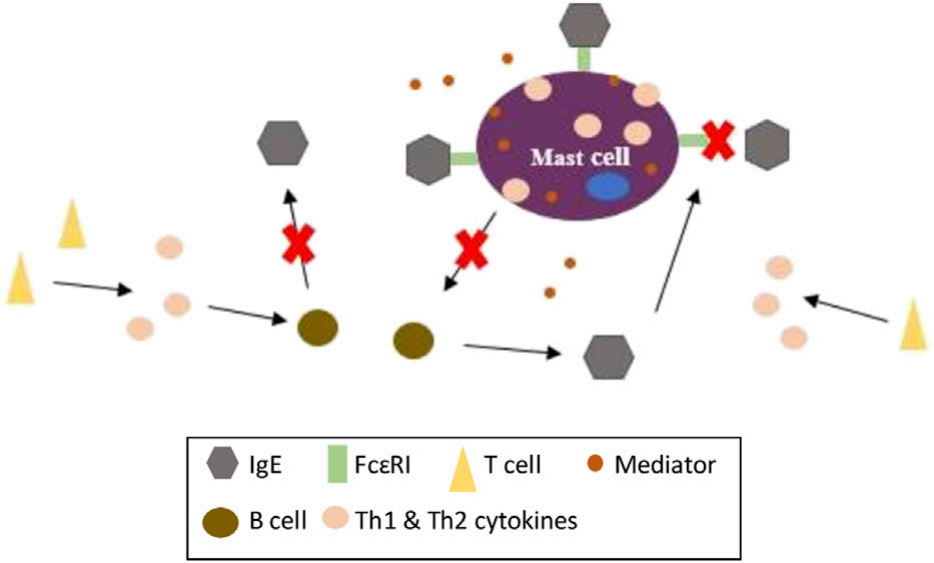
The mechanism of actions of orally and topically administered herbs and their active constituents in murine model of AD.

Throughout this systematic review, we found that serum IgE, infiltration of mast cells and the levels of Th1 and Th2 cytokines were evaluated the most in 82, 62 and 53 studies respectively. These three biomarkers are interrelated in the pathogenesis of AD as depicted in Figure 3. In brief, allergen penetration stimulates T cells to secrete cytokines (IL-4, IL-5, IL-13, IFN-γ, *etc*.). The released cytokines in turn trigger nearby B cells to produce IgE which then bind to the high affinity receptor IgE, FcɛRI on MCs. The synthesis and release of inflammatory mediators and cytokines can be induced by IgE-dependent mechanism (Williams and Galli, 2000: Kulka and Befus, 2003). The mechanism of action of these herbs and their active constituents may involve the inhibition of *1*) binding of IgE to the FcɛRI, *2*) the release of mediators and cytokines from MCs, or *3*) synthesis of IgE from B cells. Although the exact mechanism was not mentioned in each of these studies, the biomarkers of AD related to these three parameters were found to be alleviated. However, it may be difficult to compare the data from different studies as various investigators used different experimental approaches to assess the outcome of AD such as ELISA, histological analysis and dermatitis severity. Reports may also vary considerably depending on the methodological details provided that could potentially lead to risk of bias. Generally, most studies included in this systematic review did not describe their methodologies precisely which could lead to an underestimation or overestimation of the findings. Thus, it is suggested that animal experiments should be properly designed and carried out well with transparency in every step especially blinding in performance and assessment to avoid any bias. Data should be reported entirely and reasons for any dropout data should also be provided. With all the data gathered from 103 studies evaluating the efficacies of oral and topical administered herbs along with their active constituents in alleviating AD and the underlying mechanism of actions using various animal models and inducers, it is hoped that this systematic review will aid in the evidence-based selection of animal models for AD in the future.

## Conclusion

This systematic review has summarized 103 articles into several aspects based on PICO; in which the most common population (P) or choice of animal model used was BALB/C mice (*n* = 56), due to the fact that they lean towards a Th2-dominated immune response which, therefore, suit the disease of interest, AD. As for the intervention (I), this review consisted of both oral and topical administration routes of herbs and their active constituents in AD-like murine models and the most common positive control (C) drug chosen among these studies was the topical corticosteroid known as dexamethasone (*n* = 34). A substantially large number of these studies (*n* = 75) were conducted in Korea and none of the studies analysed the same herb and only two articles by Aslam et al. (2018) and Tsang et al. (2016) were understood to compare the different routes of administrations of the same herb mixture and compound. Findings from these two studies showed that both oral and topical routes of herb and their active constituents’ administration were able to alleviate AD, however, the authors did not make a distinct comparison as to which route was more effective than the other. The common outcome (O) parameters of AD assessed amongst these studies include the histological findings of AD-like skin lesions, serum IgE levels, mast cell count and Th1/Th2 cytokines. Generally, both oral and topical administration of herbs and their active constituents elicited the potential to decrease the AD parameters mentioned above. Besides, large attention was also drawn towards the production and expression of Th2 cytokines in these studies. Upon tabulation of the vast array of findings, a myriad of results obtained from majority of the oral and topical studies demonstrated that herb extracts, mixtures and compounds isolated from herbs could markedly suppress IgE levels and MCs infiltration or degranulation. Theoretically, upon successful inhibition of IgE-mediated activation of MCs, they therefore hinder the subsequent degranulation and secretion of pro-inflammatory cytokines and preformed mediators. Taken together, these findings allow us to propose that the anti-AD effects could be attained by down-regulation of IgE-mediated MCs activation in *in vivo* models. Therewithal, MAPK and NF-κB pathways are worth to dive into for future research endeavours as the inhibition of these signalling pathways have been recognized as a potential therapeutic candidate against AD. It is also important to note that majority of the studies included in this systematic review mentioned about compliance with animal welfare as well as conflict of interest statement among the authors. However, most studies did not distinctly elaborate on examiner blinding, sample size calculation and animal allocation. As a result, there is ample room for methodological improvement in these studies as the authors could declare these aspects in order to circumvent an unclear risk of bias. Therefore, future research should implement these crucial details to enhance the quality of studies.

## Data Availability

The original contributions presented in the study are included in the article/Supplementary Material, further inquiries can be directed to the corresponding author.
