# Supplementary material for: Management of Atopic Dermatitis Via Oral and Topical Administration of Herbs in Murine Model: A Systematic Review
Source: Front Pharmacol. 2022 May 24;13:785782. doi: 10.3389/fphar.2022.785782 (PMC9171034; doi:10.3389/fphar.2022.785782)
Supplement: Supplementary file 1 [file Table4.pdf]

Supplementary Table 4: The preparation and chemical analysis of the topical treatments of herbs and their active constituents

Species/compound, source, concentration	Quality control reported? (Y/N)	Chemical analysis reported? (Y/N)	Purity (%) [For compound only]	References
Whole plant of *Isodon inflexus* (Thunb.) Kudo, [CheonBuk National University, Jeonju, Korea)], 100 g<br><br>Voucher specimen (no. KIOM 53) was deposited at the herbarium of the Mibyeong Research Center at the KIOM	Y- Extracted two times in 1 L of 70% ethanol for 120 min followed by filtration through filter paper (Whatman No. 1). After filtration, specimens were concentrated under reduced pressure. Extracts were lyophilized.	Y- HPLC	N/A	(112)
Whole plant of *Gardenia jasminoides* J. Ellis, [Mega Herb, Co. (CheonBuk, Korea)], 100 g<br><br>Voucher specimen (no. KIOM 32/KIOM 51) was deposited at the herbarium of the Herbal Medicine Research Division at the KIOM	Y- Extracted in 1 L of 70% ethanol for 120 min followed by filtration. The extract was dried under reduced pressure.	Y- HPLC	N/A	(111)
Whole plant of *Morus alba* L., [HMAX (Jecheon, Korea)], 200 g<br><br>Voucher specimen (2008-ST12) has been deposited in the Basic Herbal Medicine Research Group, Korea Institute of Oriental Medicine	Y- Extracted three times with 70% ethanol by sonication for 60 min. The extract was filtered, evaporated to dryness, and freeze-dried	N	N/A	(85)
Whole plant of *Artemisia capillaris* Thunb., [Kwangmyungdang Medicinal herbs (Ulsan, Korea)], 300g	Y- Extracted with 70% EtOH by sonication for 60 min. The extract solution was filtered through Whatman No. 2 filter paper and evaporated to dryness.	Y- HPLC	N/A	(31)

Voucher specimen (AC-2009-EBM30) has been deposited at the Herbal Medicine Formulation Research Group at the Korea Institute of Oriental Medicine				
Whole plant of *Saxifraga stolonifera* (L) Meeb (Saxifragaceae), [Zunyi Jishi Pharmaceutical Co., Ltd. China], 500 g<br><br>Lot no. 20150725	Y- Pulverized to fine powder, degreased to colorless by petroleum ether and then dried, extracted twice with 60% EtOH at 80°C.	N	N/A	(143)
Leaves of *Spirodela polyrhiza* (L.) Schleid., [Jungdo Pharm. INC (Seoul, Korea)], 30 g<br><br>No voucher specimen or batch number was mentioned	Y- Shaken in 70% ethanol for 24h at room temperature. The extract was filtered, concentrated in a rotary vacuum evaporator and lyophilized.	Y- HPLC-DAD	N/A	(77)
Leaves of *Alpinia intermedia* Gagnep., [Gray Art (Tokyo, Japan)]<br><br>No information on the concentration of the herb<br><br>No voucher specimen or batch number was mentioned	Y- Chopped and boiled, and the evaporated fractions were retrieved and diluted in water.	N	N/A	(2)
Leaves of *Pyrus ussuriensis* Maxim., [Hwacheon-gun in the Gangwon-do Province of South Korea], 5 kg<br><br>Voucher Number SU-160706001 was deposited at herbarium of Sungkyunwan University, Suwon, South Korea	Y- Powdered, soaked in 70% ethanol for three days, extracted, filtered and concentrated using a rotary evaporator.	Y- HPLC	N/A	(17)
Leaves of *Pyrus ussuriensis* Maxim.	Y- 70% EtOH total extract was fractionated with	Y- HPLC	N/A	(16)

No information of the supplier and the concentration of the herb<br><br>No voucher specimen or batch number was mentioned	hexane, chloroform, ethyl acetate, n-butanol and water. The solvents were then evaporated and freeze-dried. Further, the chloroform fraction was separated using stepwise gradient elution of ethyl acetate-methanol in a silica gel column chromatography.			
Leaves of *Urtica thunbergiana* Siebold & Zucc., [Luvama Nature Co., Ltd. (Gyeonggi-do, Korea)], 5 g<br><br>No voucher specimen or batch number was mentioned	Y- Extracted using 50% ethanol in a digital orbital shaker for 24 h at 24–25°C three times. After incubation, the extract was filtered and evaporated under vacuum at 40°C.	N	N/A	(100)
Leaves of *Broussonetia kazinoki* Siebold, [Chun-Yang Paper Corporation of Jeonbuk, Province, Korea], 4.5 kg<br><br>Voucher specimen (JSI0862) was deposited at the Jeonju Biomaterials Institute	Y- Chopped into small pieces and extracted with 70% ethanol with reflux for 4 hours. The extracted solution was filtered and evaporated under reduced pressure.	N	N/A	(76)
Stems and leaves of *Wikstroemia indica* (L.) C.A.Mey., [Zobra village, Hathazari Upazila, Chittagong], 4 kg<br><br>Voucher specimen (PNU-0026) was deposited at the Medicinal Herb Garden of Pusan National University	Y- Chopping them into small millimeter-sized pieces. The plants prepared were extracted with 95% ethanol and concentrated to dryness in vacuum.	Y- HPLC/MS	N/A	(83)
Roots of *Angelica sinensis* (Oliv.) Diels, [Jungdo Herb (Seoul, Korea)], 30 g<br><br>Voucher specimen (#E70AS) was deposited in	Y- Cut into small pieces and extracted with 70% ethanol for 24h at room temperature. The filtrate was concentrated under	Y- HPLC	N/A	(78)

the herbarium of the Department of Convergence Korean Medical Science	reduced pressure using Whatman filter paper no. 3. The resultant solutions were concentrated in a rotary evaporator, freeze-dried for three days.			
Roots of *Inula helenium* L., [Anguo county, Hebei province, China]<br><br>No information on the concentration of the herb<br><br>Voucher specimen (No. 2013.09.22) was deposited in the herbarium of Second Military Medical University	N	N	N/A	(134)
Fruits of *Tribulus terrestris* L., [Medicinal Materials Company (Omniherb, Kyungsan, Korea)], 200 g<br><br>Voucher specimens (No. 2016-TF-E30) were deposited in the Herbarium of the College of Korean Medicine, Dongguk University	Y- Extracted with 30% ethanol for 3 h, filtered through Whatman Grade 1 filter paper, concentrated under a vacuum rotary evaporator at 60°C, and then lyophilized in a freeze-dryer.	Y-HPLC	N/A	(57)
Fruits of *Juniperus rigida* Siebold & Zucc., [Kyung-Dong Herb market (Seoul, Korea)], 900 g<br><br>Voucher specimen (PNU-0021) has been deposited in the Medicinal Herb Garden, Pusan National University	Y- Extracted with 95% ethanol and evaporated under reduced pressure.	Y- Q-TOF LC/MS	N/A	(82)
Fruits of *Gardenia jasminoides* J.Ellis, [Omniherb Co. (Yeoungcheon, Korea)], 250.54 g	Y- Soaked three times in 70% ethanol in an ultrasonic bath for 90 min. This residue was filtered and evaporated in vacuum.	Y- HPLC	N/A	(125)

Voucher specimen (No. 000599) was deposited in the herbarium of the Department of Herbal Resources Research at the KIOM				
Fruits of *Forsythia suspensa* (Thunb.) Vahl, [Omniherb Co. (Yeoungcheon, Korea)], 50 g<br><br>Voucher specimen (No. KIOM-78039) was deposited in the herbarium of the Department of Herbal Resources Research at the KIOM	Y- Soaked three times in 70% ethanol in an ultrasonic bath for 60 min. The ethanol supernatants were combined, filtered, and evaporated in vacuum.	Y- HPLC	N/A	(124)
Rhizomes of *Dioscorea quinquelobata* Thunb., [JirisanHanbang Food® (Sancheong, Gyeongnam, South Korea)], 20 kg<br><br>Voucher specimen (PNU-0023) has been deposited in the Medicinal Herb Garden, Pusan National University	Y- Extracted with 95% EtOH and evaporated under reduced pressure.	Y- HPLC/MS	N/A	(48)
Rhizomes of *Coptis chinensis* Franch., [Good Agricultural Practices Demonstration Base in Shizhu (Chongqing, China)], 10 kg<br><br>No voucher specimen or batch number was mentioned	Y- Pulverized and soaked with 95% ethanol at room temperature. The extract was concentrated to give a residue.	Y- HPLC & NMR	N/A	(137)
Barks of *Pterocarpus santalinus* L.f., [Kyungdong Oriental Market (Seoul, South Korea)], 1 kg<br><br>Voucher specimen (D-1016) was deposited	Y- Refluxed with boiling 50% ethanol for 3 h. The extract was filtered using Whatman No. 1 filter paper, dried under vacuum and subsequently freeze-dried.	Y- HPLC/MS	N/A	(33)

at the Korea Institute of Science and Technology (KIST)-Gangneung Institute, Gangneung, South Korea				
Aerials of *Stellera chamaejasme* L., [Yunnan province, Diqing prefecture, Xiang Ge Li Ra, China], 6.11 kg<br><br>Voucher specimen (PNU-0023) was deposited at the Medicinal Herb Garden, Pusan National University	Y- Extracted with 95% ethanol and evaporated under reduced pressure.	Y- HPLC/MS	N/A	(52)
Aerials of *Centella asiatica* (L.) Urb., 1 kg<br><br>No information of the supplier<br><br>No voucher specimen or batch number was mentioned	Y- Extracted with ethanol at room temperature. The extracts were filtrated with depth-filter coated with active carbon and concentrated at 80°C under reduced pressure.	N	N/A	(44)
Acorn shell of *Quercus mongolica* subsp. crispula (Blume) Menitsky, [Wonju, Gangwon-do area in Korea], 46.5 g<br><br>Voucher specimen (SN20151001) has been deposited at the Natural Products Research Institute of the Korea Institute of Science and Technology	Y- Extracted thrice with 95% and evaporated under vacuum.	Y- HPLC	N/A	(81)
Fruit pulps of *Cucumis melo* L., [Kwangmyungdang Medicinal herbs (Ulsan, Korea)], 300 g<br><br>Voucher specimen (2009-EBM9) has been deposited at the Herbal Medicine	Y- Extracted with 70% ethanol by sonication for 60 min. The extract solution was filtered through a filter paper and evaporated to dryness using a rotary evaporator.	N	N/A	(32)

Formulation Research Group at the Korea Institute of Oriental Medicine				
Panax ginseng C.A.Mey., [Ohki Pharmaceutical Co., Ltd. (Tokyo, Japan)], 500 g<br><br>No information on the part of the herb<br><br>No voucher specimen or batch number was mentioned	Y- Crushed and refluxed for 2 h twice in 70% methanol. The filtrate was evaporated to dryness under reduced pressure.	N	N/A	(104)
Barks of *Acer tegmentosum* Maxim., [Hamyang County, Gyeongsangnamdo, Republic of Korea], 1.2 kg<br><br>No voucher specimen or batch number was mentioned	Y- Extracted twice in methanol for 2h in a reflux apparatus. After reflux and filtration, the extract was evaporated in vacuum and lyophilized.	Y- HPLC	N/A	(140)
Rhizomes of *Cyperus rotundus* L., [oriental Medicines market (KyungDong, Seoul, Korea)], 3 kg<br><br>Voucher specimen (DKH-02561) was deposited at the Ministry of Food and Drug Safety, Korea	Y- Chopped and extracted twice with methanol under reflux for 3 h to yield the crude extract.	Y- GC-MS	N/A	(142)
Whole plants of *Persicaria tinctoria* (Aiton) Spach, [Naju city, republic of Korea]<br><br>No information on the concentration of the herb<br><br>No voucher specimen or batch number was mentioned	Y- Dipped in water for 3 days. Lime was put in the water and the water was stirred until the color was changed into indigo blue. After indigo dye sank, supernatant which was changed clearly was drained. The indigo dye sunk like mud is named Niram.	Y- HPLC	N/A	(38)
Roots of *Vincetoxicum atratum* (Bunge) C. Morren	Y- Extracted with distilled water at room temperature for 24 h. Then, the extract	N	N/A	(23)

& Decne., [Jung-do Herb Inc. (Seoul, Korea)], 100 g<br><br>Voucher specimen (CA_root_2013) was deposited at Korean Medicine at Kyung Hee University	was filtered using 0.22 mL filter and concentrated under reducing pressure.			
Roots of *Pseudostellaria heterophylla* (Miq.) Pax, [Herb Inc. (Guri, Korea)], 100 g<br><br>Voucher specimens (no. PH131201) were stored in the College of Pharmacy of Kyung Hee University	Y- Extracted with distilled water for 24 h at room temperature. Following filtration, the extracted solution was concentrated in a rotary evaporator, freeze-dried for three days.	N	N/A	(21)
Roots of *Rosa multiflora* Thunb., [Gyeongdong Market (Seoul, Korea)], 2.5 kg<br><br>Voucher specimen (RMR2008) was deposited at the herbarium of the College of Pharmacy, Chung-Ang University, Korea	Y- Extracted three times with 80% acetone at room temperature. After removing the acetone under vacuum, the residual aqueous solution was filtered and the filtrate was then concentrated.	Y- HPLC	N/A	(110)
C2RLP<br>• *Cornus officinalis* Siebold & Zucc.<br>• *Rosa multiflora* Thunb.<br>• *Lespedeza bicolor* Turcz.<br>• *Platycladus orientalis* (L.) Franco<br>• *Castanea crenata* Siebold & Zucc.<br><br>[Gyeongbuk Forest Resource Development Institute, the Republic of Korea]	Y- Dried and crushed parts of each plant (4:1:1:1:1) were boiled in 30% ethanol. The extracts were filtered with Whatman filter paper Number 1, evaporated to dryness and freeze-dried.	Y- LC/MS	N/A	(92)

No information on the concentration of the herbs<br><br>Voucher specimens (LVPPM 2001 – 2005) were deposited at Kyungpook National University, Republic of Korea				
Dangguibohyul-Tang<br>• *Astragalus mongholicus* Bunge, 7.5 g<br>• *Angelica sinensis* (Oliv.) Diels, 11.2 g<br><br>No information of the supplier<br><br>Voucher specimen (# AM001 and # AS070) was deposited in the herbarium of the College of Korean Medicine, Kyung Hee University, Republic of Korea	Y- Herbs (5:1) were extracted with 70% ethanol for 24 h. The extracts were filtered, concentrated and lyophilized then mixed well in a vortex.	Y- HPLC	N/A	(22)
Danggui Buxue Tang<br>• *Astragalus mongholicus* Bunge, 100 g<br>• *Angelica sinensis* (Oliv.) Diels, 100 g<br><br>[Gansu and Shanxi province, China]<br><br>No voucher specimen or batch number was mentioned	Y- Herbs (5:1) were soaked in water and boiled for 60 minutes. Then, extracts were centrifuged and the supernatants were lyophilized and mixed with excipients.	N	N/A	(28)
KAJD<br>• *Phellodendron amurense* Rupr.<br>• *Sesamum indicum* L.<br>• *Sophora flavescens* Aiton	N	Y- HPLC	N/A	(42)

• *Glycyrrhiza glabra* L.<br>• *Ophiopogon japonicus* (Thunb.) Ker Gawl.<br>• *Radix rehmanniae* Exsiccat<br><br>[Hanpoong Pharm and Foods Company (Jeon-ju, Korea)]<br><br>No information on the concentration and the ratio of the herbs<br><br>No voucher specimen or batch number was mentioned				
Pentaherbs<br>• *Lonicera japonica* Thunb., 5 g<br>• *Mentha canadensis* L., 2.5g<br>• *Paeonia × suffruticosa* Andrews, 5 g<br>• *Atractylodes lancea* (Thunb.) DC., 5 g<br>• *Phellodendron amurense* Rupr., 5 g<br><br>No information of the supplier<br><br>No voucher specimen or batch number was mentioned	Y- Herbs (2:1:2:2:2) were extracted by refluxing in boiling water at 100°C for 1h. Extraction was repeated twice to obtain total water crude extract. The filtered extract was freeze-dried into powder.	Y- HPLC	N/A	(131)
Huang-Lian-Jie-Du-Tang (HLJDT)<br>• *Coptis chinensis* Franch., 15 g<br>• *Scutellaria baicalensis* Georgi, 10 g	Y- Boiling these components in water three times. Then, the solution was collected and concentrated by water-evaporation.	Y- HPLC	N/A	(27)

• *Phellodendron amurense* Rupr., 10 g<br>• *Gardenia jasminoides* J. Ellis, 15 g<br><br>[Zisun Herbal Pharmaceutical Company Ltd. (China)]<br><br>No information on the ratio of the herbs<br><br>No voucher specimen or batch number was mentioned				
Si-Ni-San<br>• *Bupleurum chinense* DC., 10 g<br>• *Paeonia lactiflora* Pall., 10 g<br>• *Glycyrrhiza uralensis* Fisch. ex DC., 10 g<br>• *Citrus aurantium* L., 10 g<br><br>[Zisun Herbal Pharmaceutical Co. Ltd. (Guangzhou, Guang Dong, China)]<br><br>No voucher specimen or batch number was mentioned	Y- Herbs (1:1:1:1) were soaked in water for 30 min and then boiled for 30 min. The extract was collected and the residue was boiled in water two more times as above. Then, the extracts were pooled and concentrated.	Y- HPLC	N/A	(26)
Atofreellage<br>• *Rhus javanica* Linne, 100 g<br>• *Kochi scoparia* Schrader, 100 g<br>• *Cnidium monnieri* (L.) Cusson, 100 g<br>• Houttuynia cordata Thunb., 100 g	Y- Dried individual herbs were mixed in distilled water at 100°C and the extraction procedure was repeated with water then centrifuged for 30 min at 1500g and the supernatant was lyophilized using a vacuum-freeze-drying system.	Y- HPLC	N/A	(67)

• *Nepeta tenuifolia* Benth., 100 g<br>• *Sophora flavescens* Aiton, 100 g<br>• *Rheum palmatum* L., 100 g<br>• *Lithospermum erythrorhizon* Siebold & Zucc., 100 g<br>• *Terminalia chebula* Retz., 100 g<br>• *Trichosanthes kirilowii* Maxim., 100 g<br><br>[Jeong-Seong Oriental Pharmacy Store (Daejeon, Korea)]<br><br>No information on the ratio of the herbs<br>No voucher specimen or batch number was mentioned				
• *Aucklandia costus* Falc.<br>• *Platycladus orientalis* (L.) Franco<br><br>[Kyung-Dong Herb market (Seoul, Korea)]<br><br>No information on the concentration and the ratio of the herbs<br><br>Voucher specimens University (No. 2014-05 and No. 2014-06) were deposited at the herbarium of Department of Food & Nutrition, Hoseo University	Y- Extracted with 1, 3-butylene in an ultrasonic extractor at room temperature for 12 h and filtered. The filtrates were centrifuged and the supernatants were used.	Y- UPLC-MS/MS	N/A	(141)

Jawoongo<br>• *Arnebia euchroma* (Royle ex Benth.) I.M.Johnst.<br>• *Angelica gigas* Nakai<br><br>[Han-poong Pharm Co., Ltd. (Jeonjoo, Republic of Korea)]<br><br>No information on the concentration and the ratio of the herbs<br><br>No voucher specimen or batch number was mentioned	N	Y- HPLC	N/A	(70)
5,6-dihydroergosterol-glucoside<br><br>No information of the supplier and the concentration of the compound	N	N	N	(55)
Ophiopogonin D<br><br>[Chemfaces (Wuhan Chemfaces Biochemical Co., Ltd., China)]<br><br>No information on the concentration of the compound	N	Y-UPLC/ESI-QTOF-MS	N	(4)
Peiminine<br><br>[Abcam (Cambridge, UK)]<br><br>No information on the concentration of the compound	N	N	≥ 98%	(88)
3,5-dicaffeoyl-epi-quinic acid	N	N	N	(115)

[Korea Maritime University]<br><br>No information on the concentration of the compound				
Spilanthol<br><br>[ChromaDex, Irvine, CA, USA]<br><br>No information on the concentration of the compound	N	N	N	(46)
7,8,4'-Trihydroxyisoflavone<br><br>No information of the supplier and the concentration of the compound	N	N	N	(63)
Crocin<br><br>[Sigma Aldrich Co. (St. Louis, MO, USA)]<br>No information on the concentration of the compound	N	N	N	(123)
Stechamone<br><br>[Yunnanprovince (Diqing prefecture, Xiang Ge Li Ra, China)]<br><br>No information on the concentration of the compound	Y- Extracted with 95% ethanol and the extract obtained was then evaporated under reduced pressure, suspended in distilled water. The ethyl acetate fraction was then chromatographed to yield 13 fractions. Stechamone was obtained by recrystallization.	Y- HPLC	N	(52)
Galangin<br><br>[Sigma (St. Louis, MO)]<br><br>No information on the concentration of the compound	N	N	N	(19)
Eupatilin	N	N	N	(56)

[Phytochemicals Online (www.phytopurify.com)] No information on the concentration of the compound				
Quercetin<br><br>[Nanjing Goren Bio-Technology Co., Ltd. (Nanjing, China)]<br><br>No information on the concentration of the compound	N	N	≥ 98%	(45)
Thymoquinone<br><br>[Sigma Aldrich (US)]<br><br>No information on the concentration of the compound	N	N	≥ 98%	(5)
Pulegone<br><br>[Sigma-Aldrich Chemical Co. (MO, USA)]<br><br>No information on the concentration of the compound	N	N	N	(24)

HPLC-DAD: High-Performance Liquid Chromatography Diode Array Detector, GC-MS: Gas Chromatography-Mass Spectrometry, KIOM: Korea Institute of Oriental Medicine
