# Supplementary material for: Management of Atopic Dermatitis Via Oral and Topical Administration of Herbs in Murine Model: A Systematic Review
Source: Front Pharmacol. 2022 May 24;13:785782. doi: 10.3389/fphar.2022.785782 (PMC9171034; doi:10.3389/fphar.2022.785782)
Supplement: Supplementary file 2 [file Table1.pdf]

Supplementary Table 1: Study parameters and suggested mechanism (s) of the included oral administration of herb and their active constituents' studies.

Herb Name	Sample Type	Experimental Outcomes	Proposed Mechanism	References
Angelica gigas Nakai	Ethanolic extract (Dried whole plant)	↓dermatitis severity<br>↓serum IgE<br>↓serum IL-6<br>↓serum & tissue TNF-α<br>↓serum histamine<br>↓MAPKs phosphorylation (ERK, JNK & p38)	Suppression of both Th1 & Th2 cytokines via inhibition of MAPK & NF-κB signaling pathways	(102)
Artemisia caruifolia Buch. -Ham. ex Roxb.	Ethanolic extract (Dried whole plant)	↓dorsal skin thickness<br>↓skin lesions<br>↓ear thickness	Restoration in dorsal skin thickness and restrained edema in ear and skin<br><br>Analysis on signaling pathway was not performed	(144)
Ribes fasciculatum var. *chinense* MAX.	Ethanolic extract (Roots)	↓serum IgE	Suppression of serum IgE level<br><br>Analysis on signaling pathway was not performed	(54)
Sanguisorba officinalis L. (Rosacease)	Ethanolic extract (Roots)	↓mast cell count<br>↓serum IgE<br>↓skin eosinophils<br>↓scratching frequency<br>↓epidermal thickness	Suppression of mast cell count & IgE level<br><br>Analysis on signaling pathway was not performed	(146)
Poria cocos F.A. Wolf	Ethanolic extract (Bark)	↓dermatitis severity<br>↓tissue IL-4<br>↑tissue IL-10 (anti-inflammatory)<br>↓epidermal thickness	Suppression of one Th2 cytokine (IL-4) but enhancement of another Th2 cytokine (IL-10)	(6)

			Analysis on signaling pathway was not performed	
Salvia plebeia R. Br. (Lamiaceae)	Ethanolic extract (Dried whole plant)	↓mast cell count<br>↓serum IgE<br>↓skin eosinophils<br>↓tissue TNF-α, IFN-γ, IL-4, IL-13, IL-31 & Th17<br>↓epidermal & dermal thickness<br>↓serum histamine	Suppression of both Th1 & Th2 cytokines<br><br>Analysis on signaling pathway was not performed	(20)
Helianthus tuberosus L.	Ethanolic extracts (Tubers)	↓dermatitis severity<br>↓mast cell count<br>↓serum IgE, IL-6<br>↓tissue TNF-α<br>↓serum histamine<br>↓epidermal thickness<br>↓ICAM-1 and VCAM-1 expression<br>↑filaggrin expression	Suppression of both Th1 & Th2 cytokines and restoration of skin barrier<br><br>Analysis on signaling pathway was not performed	(58)
Tabebuia avellanedae Lorentz ex Griseb.	Ethanolic extract (N/A)	↓dermatitis severity<br>↓serum IgE, IL-4 & IFN-γ<br>↓tissue mRNA IL-4, IL-5 & IFN-γ<br>No significant changes of IL-12<br>↓mRNA IL-4, IL-5, IFN-γ & IL-12<br>↓epidermal thickness<br>↓serum histamine<br>↓Syk/NFκB pathway	Suppression of both Th1 & Th2 cytokines via inhibition of NF-κB signaling pathway	(108)
Artemisia argyi H. Lév. & Vaniot	Ethanolic extract (N/A)	↓skin lesions<br>↓serum IgE, IL-1β, IL-4, IL-6<br>↓mRNA IL-4, IL-6, IFN-γ, IL-1β, TNF-α & GM-CSF<br>↓serum histamine<br>↓tissue TGF-β1 expression<br>↓Lyn, Syk, PI3K/Akt & IκBα phosphorylation<br>↓MAPKs phosphorylation (ERK, JNK & p38)	Suppression of both Th1 & Th2 cytokines via inhibition of MAPKs signaling pathway	(34)
Persicaria tinctoria (Aiton) Spach (Naju Jjok)	Distilled water extract	↓dermatitis severity<br>↓serum IgE<br>↓mRNA TSLP, IL-4, IFN-γ<br>↓mRNA IL-6, TNF-α, TARC	Suppression of both Th1 & Th2 cytokines	(35)

	(Dried whole plant)	↓protein IL-4 & IL-6<br>↓serum IL-4<br>↓caspase-1 expression<br>No significant changes in the scratching & serum histamine	Analysis on signaling pathway was not performed	
Angelica dahurica (Hoffm.) Benth. & Hook.f. ex Franch. & Sav.	Distilled water extract (N/A)	↓mast cell count<br>↓serum IgE, IL-6, IL-10 & IL-12<br>↓CD4+ (total T cells)<br>↓number of WBCs,<br>↓mRNA IL-4, IL-6 & TNF-α	Suppression of both Th1 & Th2 cytokines<br><br>Analysis on signaling pathway was not performed	(69)
Ixeris dentata (Thunb.) Nakai	Distilled water extract (N/A)	↓serum IgE<br>↓ MAPKs phosphorylation (ERK, JNK, p38)<br>↓IκBα & NFκB phosphorylation	Inhibition of MAPKs & NF-κB signaling pathways	(49)
Panax ginseng C.A. Mey.	Distilled water extract (N/A)	↓dermatitis severity<br>↓mast cell count<br>↓serum IgE<br>↓blood eosinophils<br>↓ceramidase (enzyme)<br>↓TSLP, IL-6 & TNF-α, TARC<br>↓epidermal thickness<br>↓MAPKs phosphorylation (ERK, JNK & p38)<br>↑filaggrin levels<br>↓TEWL<br>↑skin hydration	Suppression of both Th1 & Th2 cytokines via inhibition of MAPKs signaling pathway and restoration of skin barrier	(109)
Panax ginseng Meyer	Water extract (Roots)	↓serum IgE, IL-6<br>↓tissue TARC & MDC<br>↓MAPKs phosphorylation (ERK, JNK & p38)<br>↓IkBa & NFκB expression	Suppression of Th2 cytokines via inhibition of MAPKs & NF-κβ signaling pathways	(60)
Polygala tenuifolia Willd.	Distilled water extract (Roots)	↓dermatitis severity<br>↓serum IgE<br>↓mRNA TNF-α & IL-4<br>↓scratching frequency<br>↓serum histamine<br>↓epidermal thickness	Suppression of both Th1 & Th2 cytokines<br><br>Analysis on signaling pathway was not performed	(126)
Liriope muscari (Decne.) L.H. Bailey	Water extract (Roots)	↓mast cell count<br>↓serum IgE<br>↓epidermal thickness	Suppression of mast cell count & serum IgE level	(72)

		↓ear thickness<br>↓lymph nodes weight	Analysis on signaling pathway was not performed	
Saussurea lappa (Decne.) C.B. Clarke	Methanolic extract (Dried whole plant)	↓mRNA CCR1, IL2Rβ<br>↓mRNA PI3Kca, IL20Rβ	Suppression of chemokine (CCR1), interleukins (IL2Rβ, IL20Rβ) & enzyme (PI3Kca)<br><br>Analysis on signaling pathway was not performed	(87)
Saussurea lappa (Decne.) C.B. Clarke	Methanolic extract (Dried whole plant)	↓dermatitis severity<br>↓mast cell count<br>↓serum IgE<br>↓plasma TARC<br>↓epidermal hyperplasia	Suppression of mast cell count, serum IgE & chemokine (TARC)<br><br>Analysis on signaling pathway was not performed	(86)
Dendrobium officinale Kimura & Migo	Ethyl acetate extract (Stems)	↓mast cell count<br>↓serum IgE<br>↓epidermal thickness<br>↓tissue IL-4<br>↑tissue IFN-γ<br>No significant changes of IL-6	Suppression of Th2 cytokines and elevation of Th1 cytokines<br><br>Analysis on signaling pathway was not performed	(137)
Patrinia scabiosifolia Link	DMSO extract (Dried whole plant)	↓dermatitis severity<br>↓serum IgE, AST & ALT<br>↓tissue IL-4, IL-5, IL-13<br>↓eotaxin<br>↓epidermal atrophy, hypertrophy<br>↓hyperkeratosis<br>↑filaggrin expression<br>↓JNK1, JNK2 & NF-κB phosphorylation	Suppression of Th2 cytokines via inhibition of NF-κB signaling pathway and restoration of skin barrier	(13)
Byakkokakeishito<br>• Gypsum (natural hydrous calcium sulfate)	Mixture (Water extracts)	↓serum IgE<br>↓ear swelling	Suppression of IgE-mediated triphasic skin reaction	(90)

• Rhizome of *Anemarrhena asphodeloides* Bunge<br>• Bark of the trunk of *Cinnamomum verum* J. Presl<br>• Seed of *Oryza sativa* L.<br>• Root and stolon of *Glycyrrhiza uralensis* Fisch. ex DC.			Analysis on signaling pathway was not performed	
Qingre-Qushi<br>• *Hedyotis diffusa* Willd.<br>• *Taraxacum* (no specific species was mentioned)<br>• *Xanthium sibiricum* Patrin ex Widder<br>• *Sophora flavescens* Aiton	Mixture (Water extracts)	↓dermatitis severity<br>↓serum IgE<br>No significant changes in IFN-γ & TNF-α<br>↓mRNA IL-4 & IL-13<br>↓tissue IL-33 & ST2 (its receptor)<br>↓scratching frequency<br>↓epidermal thickness	Suppression of Th2 cytokines<br><br>Analysis on signaling pathway was not performed	(14)
Yu-Ping-Feng-San<br>• *Astragalus mongholicus* Bunge<br>• *Atractylodes macrocephala* Koidz.<br>• *Saposhnikovia divaricata* (Turcz. Ex Ledeb.) Schischk.	Mixture (Water extracts)	↓ear swelling<br>↓serum IgE, IL-4 ↓, IL-5 ↓, IL-13<br>No significant changes in tissue IFN-γ	Suppression of Th2 cytokines<br><br>Analysis on signaling pathway was not performed	(148)
Yu-Ping-Feng-San<br>• *Astragalus mongholicus* Bunge<br>• *Atractylodes macrocephala* Koidz.	Mixture (Water extracts)	↓tissue IL-4, IL-5, IL-9, IL-13<br>↓protein & mRNA TSLP & IL-33<br>↓ear swelling	Suppression of Th2 cytokines<br><br>Analysis on signaling pathway was not performed	(138)

• *Saposhnikovia divaricata* (Turcz. ex Ledeb.) Schischk.				
Pentaherbs<br>• *Lonicera japonica* Thunb.<br>• *Mentha canadensis* L.<br>• *Paeonia suffruticosa* Andrews<br>• *Atractylodes lancea* (Thunb.) DC.<br>• *Phellodendron amurense* Rupr.	Mixture (Water extracts)	↓mast cell count<br>↓serum IgE<br>↓skin eosinophils<br>↓serum IL-4 ↓, IL-5 ↓, IL-12<br>↓epidermal thickness	Suppression both Th1 & Th2 cytokines<br><br>Analysis on signaling pathway was not performed	(131)
Hataedock<br>• *Glycine max* (L.) Merr.<br>• *Artemisia caruifolia* Buch.-Ham. ex Roxb.<br>• *Morus alba* L.	Mixture (Water extracts)	↓dermatitis severity<br>↓epidermal thickness<br>↑levels of filaggrin, involucrin & loricrin<br>↓papillary dermal edema	Restoration in lipid barrier formation and epidermal structures & suppression of epidermal inflammation<br><br>Analysis on signaling pathway was not performed	(64)
Hataedock<br>• *Coptis japonica* (Thunb.) Makino,<br>• *Glycyrrhiza uralensis* Fisch. ex DC.	Mixture (Water extracts)	↓dermatitis severity<br>↓angiogenesis<br>↓epithelial hyperplasia<br>↑filaggrin<br>↑liver X factor (LXR)<br>↓protein kinase C (PKC)<br>↓FcεRI, Substance P, Matrix metalloproteinases (MMP)-9 & 5- hydroxytryptamine (HT)<br>↓tissue IL-4, IL-13, STAT-6 & CD40+<br>↓NFκB p65, p-IκB & iNOS	Suppression of Th2 cytokines via inhibition of NF-κB signaling pathway and restoration of skin barrier	(12)
Hataedock	Mixture	↓dermatitis severity<br>↓protein kinase C (PKC)	Suppression of Th2 cytokines and	(11)

• *Coptis chinensis* Franch.<br>• *Glycyrrhiza uralensis* Fisch. ex DC.	(Water extracts)	↑liver X factor (LXR)<br>↓MMP & Substance P<br>↓tissue IL-4<br>↓mast cell degranulation<br>↓epidermal hyperplasia<br>↓tissue TNF-α, p-IκB, iNOS & COX-2	restoration of skin barrier	
Soshiho-tang<br>• *Bupleurum falcatum* Linne<br>• *Pinellia ternate* Breitenbach<br>• *Zingiber officinale* Roscoe<br>• *Scutellaria baicalensis* Georgi<br>• *Panax ginseng* C.A. Meyer<br>• *Glycyrrhiza uralensis* Fischer<br>• *Zizyphus jujube* Miller var. *inermis* Rehder	Mixture (Distilled water extracts)	↓serum IgE,<br>↓serum IL-4<br>↓tissue TNF-α, IL-1β & IL-6<br>↓epidermal & dermal thickness<br>↓ICAM-1 & NFκB expressions	Suppression of both Th1 & Th2 cytokines via inhibition of NF-κB signaling pathway	(79)
Gamisasangja-tang<br>• *Stemona sessilifolia* (Miq.) Miq.<br>• *Spirodela polyrhiza* (L.) Schleid.<br>• *Cnidium monnieri* (L.) Cusson<br>• *Sophora flavescens* Aiton<br>• *Angelica gigas* Nakai<br>• *Clematis terniflora* var. mandshurica (Rupr.) Ohwi	Mixture (Distilled water extracts)	↓dermatitis severity<br>↓hyperkeratosis & hemorrhage<br>↓mast cell count<br>↓plasma IgE<br>↓total leukocytes<br>↓tissue IL-31, IL-4, IL-5, IL-13<br>↓plasma eotaxin & TARC	Suppression of Th2 cytokines<br><br>Analysis on signaling pathway was not performed	(106)

SSC201<br>• *Stemona japonica* (Blume) Miq.<br>• *Spirodela polyrhiza* (L.) Schleid.<br>• *Cnidium monnieri* (L.) Cusson	Mixture (Distilled water extracts)	↓dermatitis severity<br>↓mast cell count<br>↓serum IgE<br>↓blood eosinophils<br>↓serum eotaxin & TARC<br>↓tissue IL-4, IL-5 & IL-13<br>No significant changes of IFN-γ<br>↓CD4+ & CCR3+<br>↓scratching frequency<br>↓epidermal & dermal thickness	Suppression of Th2 cytokines<br><br>Analysis on signaling pathway was not performed	(105)
CP001<br>• *Houttuynia cordata* Thunb<br>• *Rehmannia glutinosa* (Gaertn.) DC.<br>• *Betula platyphylla* var. japonica<br>• *Rubus coreanus* Miq.	Mixture (Ethanolic extracts)	↓mast cell count<br>↓serum IgE<br>↓mRNA IL-4 & IL-13	Suppression of Th2 cytokines<br><br>Analysis on signaling pathway was not performed	(66)
Huang-Lian-Jie-Du<br>• *Coptis chinensis* Franch.<br>• *Scutellaria baicalensis* Georgi<br>• *Phellodendron chinense* C.K. Schneid.<br>• *Gardenia jasminoides* J. Ellis	Mixture (Ethanolic extracts)	↓dermatitis severity<br>↓mast cell count<br>↓serum IgE, IL-4, TNF-α & TSLP<br>↓epidermal thickness<br>↓serum histamine<br>↓MAPKs phosphorylation (p38, JNK and ERK1/2)	Suppression of both Th1 & Th2 cytokines via inhibition of MAPKs & NF-κB signaling pathways	(15)
Sanpaocao<br>• *Cardiospermum halicacabum* L.<br>• *Physalis angulata* L.<br>• *Ludwigia adscendens* (L.) H. Hara	Mixture (Ethanolic extracts)	↓serum IgE<br>↓ear swelling & thickening	Suppression of serum IgE level<br><br>Analysis on signaling pathway was not performed	(121)

BuShenYiQi<br>• *Astragalus mongholicus* Bunge<br>• *Epimedium sagittatum* (Siebold & Zucc.) Maxim<br>• *Rehmannia glutinosa* (Gaertn.) DC.	Mixture (N/A)	↓dermatitis severity<br>↓mast cell count<br>↓serum IgE, IL-4 & IL-5<br>↑serum IFN-γ<br>↓epidermal and dermal thickness<br>↓lymphocyte count<br>↓serum ACTH (hormone) & corticosterone	Suppression of Th2 cytokines and Elevation of Th1 cytokine<br><br>Analysis on signaling pathway was not performed	(68)
Yupingfeng<br>• *Astragalus mongholicus* Bunge<br>• *Atractylodes macrocephala* Koidz.<br>• *Saposhnikovia divaricata* (Turcz. ex Ledeb.) Schischk.	Mixture (N/A)	↓EASI (dermatitis severity)<br>↓epidermal thickness<br>↓TEWL<br>↓ protein AQP3	Acceleration of wound healing and restoration of skin barrier via down regulation of AQP3 (membrane transporter)<br><br>Analysis on signaling pathway was not performed	(147)
PTQX<br>• *Atractylodes macrocephala* Koidz.<br>• *Pseudostellaria heterophylla* (Miq.) Pax<br>• *Dioscorea oppositifolia* L.<br>• *Coix lacryma-jobi* var. ma-yuen (Rom.Caill.) Stapf<br>• *Imperata cylindrica* (L.) P. Beauv.<br>• *Forsythia suspensa* (Thunb.) Vahl	Mixture (N/A)	↓dermatitis severity<br>↓ear thickness<br>↓mast cell count<br>↓serum IgE<br>↓epidermal thickness<br>↓scratching frequency<br>↓CD4+ proliferation	Suppression of mast cell count & serum IgE level<br><br>Analysis on signaling pathway was not performed	(139)

• *Dictamnus dasycarpus* Turcz.<br>• Margarita, 0.3g (no specific species)<br>• *Glycyrrhiza glabra* L.				
Taeumjowi-tang<br>• *Coix lacryma-jobi* var. ma-yuen (Rom.Caill.) Stapf<br>• *Castanea crenata* Siebold & Zucc.<br>• *Raphanus raphanistrum* subsp. sativus (L.) Domin<br>• *Schisandra chinensis* (Turcz.) Baill.<br>• *Platycodon grandiflorus* (Jacq.) A. DC.<br>• *Acorus gramineus* Aiton<br>• *Ephedra sinica* Stapf<br>• *Liriope muscari* (Decne.) L.H. Bailey	Mixture (N/A)	↓dermatitis severity<br>↓mast cell count<br>↓serum IgE<br>↓tissue eosinophils<br>↓tissue IL-4, TNF-α, IL-6<br>↓mRNA IL-4, IL-6 & TNF-α<br>↓scratching frequency<br>↓ NF-κB & MAPKs phosphorylation (ERK, JNK & p38)	Suppression of both Th1 & Th2 cytokines via inhibition of NF-κB & MAPKs signaling pathways	(107)
Jianpi Chushi<br>• *Poria cocos* F.A. Wolf<br>• *Dioscorea oppositifolia* L.<br>• *Dictamnus dasycarpus* Turcz.	Mixture (N/A)	↓serum IL-4<br>↑serum IL-2<br>↓CD4+ count<br>↓ear thickness & weight	Suppression of Th2 cytokine and elevation of Th1 cytokine<br><br>Analysis on signaling pathway was not performed	(133)

• *Zaocys dhumnade* (no information in Kewscience)<br>• *Atractylodes lancea* (Thunb.) DC.<br>• *Citrus aurantium* L.<br>• *Scutellaria baicalensis* Georgi<br>• *Sophora flavescens* Aiton<br>• *Paeonia lactiflora* Pall.<br>• *Glycyrrhiza glabra* L.				
Astragalus mongholicus Bunge	Calycosin (isolated compound)	↓mRNA IL-33 & TSLP<br>↓tissue IL-33 & TSLP<br>↓NFκB expression<br>↑occludin & ZO-1 protein	Suppression of TSLP/IL-33 via inhibition of NF-κB signaling pathway	(129)
Rheum officinale Baill.	Physcion (isolated compound)	↓dermatitis severity<br>↓mast cell count<br>↓serum IgE<br>↓serum TSLP, IL-4, IL-6 & TNF-α<br>↓protein & mRNA TSLP, IL-4 & IL-6	Suppression of both Th1 & Th2 cytokines<br><br>Analysis on signaling pathway was not performed	(96)
Rumex crispus L.	Chrysophanol (isolated compound)	↓dermatitis severity<br>↓mast cell count<br>↓serum IgE<br>↓blood eosinophil<br>↓serum TSLP, IL-4, IL-6, IL-13 & TNF-α<br>↑serum IFN-γ<br>↓epidermal thickness<br>↓serum histamine<br>↓scratching duration	Suppression of Th2 cytokines and elevation of Th1 cytokines<br><br>Analysis on signaling pathway was not performed	(37)
Saposhnikovia divaricata (Turcz. ex Ledeb.) Schischk.	Cimifugin (isolated compound)	↓ear thickness<br>↓mRNA & protein TSLP, IL-4, IL-5, IL-9 & IL-13 & IL-33<br>No significant changes of IFN-γ<br>↑CLDND1, CLDN-1 & occludin proteins	Suppression of Th2 cytokines<br><br>Analysis on signaling pathway was not performed	(135)

Fraxinus chinensis subsp. rhynchophylla (Hance) A.E. Murray	Esculetin (isolated compound)	↓mast cell count<br>↓serum IgE<br>↓tissue eosinophil<br>↓TNF-α, IFN-γ, IL-4, IL-13,<br>↓IL-31, IL-17A<br>↓epidermal & dermal thickness<br>↓serum histamine<br>↓scratching frequency	Suppression of both Th1 & Th2 cytokines<br><br>Analysis on signaling pathway was not performed	(50)
Paeonia × suffruticosa Andrews	Paeonol (isolated compound)	↓dermatitis severity<br>↓mast cell count<br>↓serum IgE<br>↓tissue IL-4 & IFN-γ<br>↓serum IL-4<br>↓mRNA & protein IL-4, IL-13<br>↓scratching frequency<br>↓serum histamine<br>↓epidermal thickness<br>↓MAPKs phosphorylation (p38 & ERK)	Suppression of both Th1 & Th2 cytokines via inhibition of MAPKs signaling pathway	(94)
Panax ginseng C.A. Mey.	Gintonin (isolated compound)	↓dermatitis severity<br>↓ear thickness<br>↓mast cell count<br>↓serum IgE, IL-4, IFN-γ<br>↓skin eosinophil<br>↓serum histamine<br>↓serum autotaxin<br>↓epidermal thickness	Suppression of both Th1 & Th2 cytokines<br><br>Analysis on signaling pathway was not performed	(75)
Persicaria tinctoria (Aiton) Spach	Tryptanthrin (isolated compound)	↓dermatitis severity<br>↓serum IgE<br>↓mRNA TSLP, IL-4, IFN-γ, IL-6, TNF-a & TARC<br>↓protein IL-4 & IL-6<br>↓serum IL-4<br>↓scratching frequency<br>↓epidermal thickness<br>↓serum histamine	Suppression of both Th1 & Th2 cytokines<br><br>Analysis on signaling pathway was not performed	(36)
Nigella sativa L.	Thymoquinone (isolated oil)	↓ear clinical score (dermatitis severity)<br>↓ear thickness & weight<br>↓total leukocyte count<br>↓serum IgE<br>↓mRNA IL-4, IL-5 & IFN-γ	Suppression of both Th1 & Th2 cytokines	(5)

		Analysis on signalling pathway was not performed

N/A: Part of the plant or type of extract was not mentioned
