# Supplementary material for: Management of Atopic Dermatitis Via Oral and Topical Administration of Herbs in Murine Model: A Systematic Review
Source: Front Pharmacol. 2022 May 24;13:785782. doi: 10.3389/fphar.2022.785782 (PMC9171034; doi:10.3389/fphar.2022.785782)
Supplement: Supplementary file 3 [file Table2.pdf]

Supplementary Table 2: The preparation and chemical analysis of the oral treatments of herbs and their active constituents

Species/compound, source, concentration	Quality control reported? (Y/N)	Chemical analysis reported? (Y/N)	Purity (%) [For compound only]	References
Whole plant of *Angelica gigas* Nakai, [Ginbu GAP Farming Corp], 330 g<br><br>No voucher specimen or batch number was mentioned	Y- Air-dried and extracted with 95% ethanol at 80°C for 4 h using the machine to obtain the extract, which was then concentrated. The extract was collected, concentrated again using a rotary vacuum evaporator.	N	N/A	(102)
Whole plant of *Artemisia caruifolia* Buch. -Ham. ex Roxb., [Yeongcheon Oriental Herbal Market], 30 g<br><br>No voucher specimen or batch number was mentioned	Y- Extracted with 70% ethanol in a 40°C shake-incubator for 24 h and filtered. The freeze-dried extract powder was dissolved in dimethyl sulfoxide and centrifuged at 14,000 rpm for 10 min. The resulting supernatant was filtered.	Y- HPLC-UV/VIS	N/A	(144)
Roots of *Ribes fasciculatum* var. *chinense* MAX., [Human herb (Gyeongbuk, Korea)], 100 g<br><br>No voucher specimen or batch number was mentioned	Y- Chopped with 70% ethanol solution under room temperature for 24 h and then filtered on a water bath under vacuo, frozen and lyophilized to yield ethanol extracts.	Y- HPLC	N/A	(54)
Roots of *Sanguisorba officinalis* L. (Rosacease), [Yeongcheon Oriental Herbal Market], 50 g<br><br>No voucher specimen or batch number was mentioned	Y- Extracted using 70% ethanol in a 40°C shaking incubator for 24 h.	N	N/A	(146)

Barks of *Poria cocos* F.A. Wolf, [Plant Extract Bank of Korea]<br><br>No information on the concentration of the herb<br><br>No voucher specimen or batch number was mentioned	Y- Extracted using 95% ethanol.	N	N/A	(6)
Whole plants of *Salvia plebeia* R. Br. (Lamiaceae), [herbal store in Jeongeup, Korea], 30 kg<br><br>Voucher specimen (number WSP-11-114) was deposited at the Herbarium of the College of Pharmacy, Woosuk University	Y- Ground and extracted with 95% ethanol for 2 weeks at room temperature. The ethanol extract was then concentrated with a rotary evaporator under reduced pressure to give a dried residue.	Y- HPLC	N/A	(20)
Tubers of *Helianthus tuberosus* L., [Yangwonfood (Cheonan, Korea)]<br><br>No information on the concentration of the herb<br><br>No voucher specimen or batch number was mentioned	Y- Extracted with 30% ethanol then filtered, lyophilized, and stored at 4°C.	N	N/A	(58)
Tabebuia avellanedae Lorentz ex Griseb., [Nutribiotech Co. Ltd. (Seoul, Korea)]<br><br>No information on the part of the herb and the concentration of the herb<br><br>No voucher specimen or batch number was mentioned	N	N	N/A	(108)

Artemisia argyi H. Lév. & Vaniot, [Omniherb Co., Ltd. (Daegu, South Korea)], 100 g<br><br>No information on the part of the herb<br><br>Voucher specimen (DKMP-201203-AAFE) was deposited at Korean Medical Physiology Laboratory, Dong-Eui University	Y- Mixed with 75% ethanol at 60˚C, incubated for 24 h and filtered.	N	N/A	(34)
Whole plant of *Persicaria tinctoria* (Aiton) Spach, [cultivated traditionally in Naju, republic of Korea]<br><br>No information on the concentration of the herb<br><br>No voucher specimen or batch number was mentioned	Y- Extracted with distilled water at 80°C for 3 h and filtered. The filtered extracts were lyophilized and reduced to powder.	Y- HPLC	N/A	(35)
Angelica dahurica (Hoffm.) Benth. & Hook.f. ex Franch. & Sav., [Han-poong Pharm Co., Ltd (Jeonjoo, Republic of Korea)]<br><br>No information on the part of the herb and the concentration of the herb<br><br>No voucher specimen or batch number was mentioned	N	N	N/A	(69)
Ixeris dentata (Thunb.) Nakai (Compositae), [Wonkwang Institute of Biomedical Engineering Research]	Y- Decocting the herbs with distilled water for 3 h and filtered and kept at 4°C.	N	N/A	(49)

No information on the part of the herb and the concentration of the herb<br><br>Voucher specimen (No. 05-17-12) was deposited at the Herbarium of the College of Pharmacy, Wonkwang University				
Panax ginseng C.A. Mey., [Gansando agriculture cooperation, Jinan, Republic of Korea], 5 g<br><br>No information on the part of the herb<br>No voucher specimen or batch number was mentioned	Y- Extracted with distilled water using a reflux condenser. The extract was then filtered and condensed under a vacuum.	N	N/A	(109)
Roots of *Panax ginseng* Meyer, [Korea Ginseng Corporation (Daejeon, Korea)]<br><br>No information on the concentration of the herb<br><br>No voucher specimen or batch number was mentioned	Y- Steaming fresh ginseng at 90-100°C for 3 h and then drying at 50-80°C, extracted with water for 8 h.	Y- HPLC	N/A	(60)
Roots of *Polygala tenuifolia* Willd. [Oriental drug store (Dongwoodang Pharmacy Co., Ltd., Yeongcheon, Republic of Korea)], 120 g<br><br>Voucher specimen (No. D0801130PTW) was deposited at the herbarium located in the college of Korean	Y- Immersed in distilled water, heated at 100°C, concentrated using a rotary evaporator and lyophilized using a freeze dryer.	Y- UPLC– ESI–MS	N/A	(126)

Medicine, Kyung Hee University				
Roots of *Liriope muscari* (Decne.) L.H. Bailey [plantations in the Miryang area (Korea)], 200 g<br><br>Voucher specimens of LP (WPC-11-010) were deposited in the Functional Materials Bank of the PNU-Wellbeing RIS Center at Pusan National University	Y- Dry roots were steamed at 99℃ for 3 h, air-dried at 70℃ for 24 h then reduced to powder, distilled water was added to the powder and the aqueous extracts were purified for 2 h at 100℃.	Y- HPLC	N/A	(72)
Dried whole plant of *Saussurea lappa* (Decne.) C.B. Clarke, [HMAX (Jecheon, Korea)], 100 g<br><br>Voucher specimen (2009-KIOM62) has been deposited in the K-herb Research Center, Korea Institute of Oriental Medicine	Y- Extracted three times with 70% methanol (1 L) by refluxing for 90 min, then filtered, evaporated to dryness, and freeze-dried.	N	N/A	(87)
Dried whole plant of *Saussurea lappa* (Decne.) C.B. Clarke, [HMAX (Jecheon, Korea)], 100 g<br><br>Voucher specimen (2009-KIOM62) has been deposited in the K-herb Research Center, Korea Institute of Oriental Medicine	Y- Extracted three times with 70% methanol (1 L) by refluxing for 90 min, then filtered, evaporated to dryness, and freeze-dried.	N	N/A	(86)
Stems of *Dendrobium officinale* Kimura & Migo, [green house of Chaoyang University ofTechnology]	Y- Dried stems of was grinded into fine powder, dissolved in methanol and sonicated at an amplitude of 15 for 10 min.	Y- LC/MS	N/A	(137)

No information on the concentration of the herb<br><br>Voucher specimen number: (CMC DT 0303) has been deposited at the Institute of Chinese Pharmaceutical Sciences, China Medical University				
Dried whole plant of *Patrinia scabiosifolia* Link, [Korea Plant Extract Bank, Korea Research Institute of Bioscience and Biotechnology (Deajeon, Korea)], 33 g<br><br>Voucher specimens (031 – 037) were deposited at the herbaria of the Department of the Herbal Pharmaceutical Development, Korea Institute of Oriental Medicine, Daejeon, Korea	Y- Dried, powdered, and then underwent extraction with DMSO for 1 day at room temperature.	N	N/A	(13)
Byakkokakeishito<br>• Gypsum (natural hydrous calcium sulfate), lot no. 7H16 M<br>• Rhizome of *Anemarrhena asphodeloides* Bunge, 0E22<br>• Bark of the trunk of *Cinnamomum verum* J. Presl, 6K20 M<br>• Seed of *Oryza sativa* L., 5G29	Y- Cutting or crushing of the whole crude drugs, boiled in 600 ml of distilled water for 60 min. The crude drugs were then removed from the paper bags and the decoctions were lyophilized.	Y- HPLC	N/A	(90)

[all purchased from Daiko Shoyaku (Nagoya, Japan)]<br><br>• Root and stolon of *Glycyrrhiza uralensis* Fisch. ex DC., [Tsumura (Tokyo, Japan)], 23040711<br><br>No information on the concentration and ratio of the herbs				
Qingre-Qushi<br>• *Hedyotis diffusa* Willd.<br>• *Taraxacum* (no specific species was mentioned)<br>• *Xanthium sibiricum* Patrin ex Widder<br>• *Sophora flavescens* Aiton<br><br>[Shuguang Hospital Affiliated with the Shanghai Chinese Medicine University]<br><br>No information on the concentrations of each herb<br><br>No voucher specimen or batch number was mentioned	Y- The four herbs (4:4:1:4) were soaked in water and boiled at 100°C twice, 1 h each time then filtered through a Whatman number 2 filter paper, concentrated under vacuum conditions, and freeze-dried.	Y- HPLC	N/A	(14)
Yu-Ping-Feng-San<br>• *Astragalus mongholicus* Bunge; [Zhangzhou Fushun Pharmaceutical	Y- Herbal mixture (RA: RAM: RS in a 3:1:1) were extracted twice with boiling water and further evaporated to crude extracts.	Y- HPLC	N/A	(148)

Co., Ltd. (Inner Mongolia, China)]<br><br>• *Atractylodes macrocephala* Koidz. [Shanxi Wanhui Pharmaceutical Co., Ltd. (Zhejiang, China)]<br><br>• *Saposhnikovia divaricata* (Turcz. ex Ledeb.) Schischk., [Herbal Decoction Slices division of Nanjing Pharmaceutical Company (Heilongjiang, China)]<br><br>No information on the concentrations of each herb<br><br>No voucher specimen or batch number was mentioned				
Yu-Ping-Feng-San<br>• *Astragalus mongholicus* Bunge; [Zhangzhou Fushun Pharmaceutical Co., Ltd. (Inner Mongolia, China)], 20150601	Y- Immersed (3:1:1) in ethanol for 1 h and then refluxed for 2 h. The extraction process was repeated twice and the extracts were combined, filtered, and evaporated to dryness using a vacuum concentrator system.	Y- HPLC	N/A	(138)

• *Atractylodes macrocephala* Koidz., [Shanxi Wanhui Pharmaceutical Co., Ltd. (Zhejiang, China)], 150102<br>• *Saposhnikovia divaricata* (Turcz. ex Ledeb.) Schischk., [Herbal Decoction Slices division of Nanjing Pharmaceutical Company (Heilongjiang, China)], 20150601<br><br>No information on the concentrations of each herb				
Pentaherbs<br>• *Lonicera japonica* Thunb., 5 g<br>• *Mentha canadensis* L., 2.5 g<br>• *Paeonia suffruticosa* Andrews, 5 g<br>• *Atractylodes lancea* (Thunb.) DC., 5 g<br>• *Phellodendron amurense* Rupr., 5 g<br><br>No information of the supplier and the ratio of the herbs	Y- Extracted by refluxing in boiling water at 100°C for 1h. Extraction was repeated twice to obtain total water crude extract. The filtered extract was freeze-dried into powder.	Y- HPLC	N/A	(131)

No voucher specimen or batch number was mentioned				
Hataedock<br>• *Glycine max* (L.) Merr., [Namyoung Pharm (Muju, Republic of Korea)], 100 g<br>• *Artemisia caruifolia* Buch. - Ham. ex Roxb.<br>• *Morus alba* L.<br><br>No information of the supplier and the concentration of the herbs<br><br>No voucher specimen or batch number was mentioned	Y- Herbs (1:1) were boiled for 3 hours in distilled water and then filtered, concentrated by using a rotatory vacuum evaporator and then freeze-dried to obtain the extract.	Y- HPLC	N/A	(64)
Hataedock<br>• *Coptis japonica* (Thunb.) Makino, [Omniherb (Yeongcheon, Korea)], 100 g<br>• *Glycyrrhiza uralensis* Fisch. ex DC., [Omniherb (Yeongcheon, Korea)], 100 g	Y- Herbs (1:1) were powdered and boiled for 3 h in distilled water then the resulting extract was filtrated, concentrated and freeze-dried.	Y- HPLC	N/A	(12)
Hataedock<br>• *Coptis chinensis* Franch., 100 g<br>• *Glycyrrhiza uralensis* Fisch. ex DC., 100 g<br><br>No information of the supplier	Y- Herbs (1:1) were decocted in distilled water for 3 hours and then filtered; after concentrating, the filtrate was freeze-dried.	Y- HPLC	N/A	(11)

No voucher specimen or batch number was mentioned				
Soshiho-tang<br>• *Bupleurum falcatum* Linne<br>• *Pinellia ternate* Breitenbach<br>• *Zingiber officinale* Roscoe<br>• *Scutellaria baicalensis* Georgi<br>• *Panax ginseng* C.A. Meyer<br>• *Glycyrrhiza uralensis* Fischer<br>• *Zizyphus jujube* Miller var. *inermis* Rehder<br><br>[Omniherb (Korea) and HMAX (China)]<br><br>Voucher specimens (2008–KE26–1 ~ KE26–7) have been deposited at the K-herb Research Center, Korea Institute of Oriental Medicine<br><br>No information on concentration and ratio of the herbs	Y- Extracted in distilled water at 100°C for 120 min, the extract was filtered through a standard sieve, evaporated and freeze-dried into powder.	Y- UPLC	N/A	(79)
Gamisasangja-tang<br>• *Stemona sessilifolia* (Miq.) Miq.<br>• *Spirodela polyrhiza* (L.) Schleid.<br>• *Cnidium monnieri* (L.) Cusson	Y- Boiling the herbs (1:1:1:1:0.5:0.5) in distilled water at 100°C for 2h. The boiled herbs were then filtered through a Whatman no.2 filter, concentrated under vacuum conditions, and freeze-dried.	Y- HPLC	N/A	(106)

• *Sophora flavescens* Aiton<br>• *Angelica gigas* Nakai<br>• *Clematis terniflora* var. mandshurica (Rupr.) Ohwi<br><br>[Dong Kyung Pharm Co., Ltd. (Seoul, Korea)]<br><br>Voucher specimens (No. 2014-004 to 009) were deposited at the College of Oriental Medicine, Daejeon University, Daejeon, Korea<br><br>No information on concentration of the herbs				
SSC201<br>• *Stemona japonica* (Blume) Miq.<br>• *Spirodela polyrhiza* (L.) Schleid.<br>• *Cnidium monnieri* (L.) Cusson<br><br>[Nanum Pharm Co., Ltd. (Yeongcheon, Gyeongbuk, Korea)]<br><br>No information on concentration of the herbs<br><br>No voucher specimen or batch number was mentioned	Y- Boiling the three herbs (1:1:1) in distilled water at 100°C for 2 h. The boiled herbs were then filtered through a Whatman no. 2 filter, concentrated under vacuum conditions and freeze-dried.	Y- HPLC	N/A	(105)
CP001<br>• *Houttuynia cordata* Thunb	Y- 30% ethanol extracted brown-colored powder.	Y- HPLC	N/A	(66)

• *Rehmannia glutinosa* (Gaertn.) DC.<br>• *Betula platyphylla* var. japonica<br>• *Rubus coreanus* Miq.<br><br>[Hanpoong Pharmaceutical (Jeon-ju, Korea)]<br><br>No information on concentration and ratio of the herbs<br><br>No voucher specimen or batch number was mentioned				
Huang-Lian-Jie-Du<br>• *Coptis chinensis* Franch.<br>• *Scutellaria baicalensis* Georgi<br>• *Phellodendron chinense* C.K. Schneid.<br>• *Gardenia jasminoides* J. Ellis<br><br>[Zhixin Herbal Pharmaceutical Company Ltd]<br><br>Voucher specimen (no. AD01–04) were deposited in the Herbarium of the School of Chinese Medicine, CUHK	Y- Herbs (3:2:2:3) were ground to powder or pieces and mixed. The mixture was extracted with 80% aqueous ethanol in an ultrasonic bath for 30 min and then was filtered.	Y- HPLC	N/A	(15)

No information on concentration of the herbs				
Sanpaocao<br>• *Cardiospermum halicacabum* L., 200 g<br>• *Physalis angulata* L., 200 g<br>• *Ludwigia adscendens* (L.) H. Hara, 200 g<br><br>[Haikou, Hainan Province, China]<br><br>No voucher specimen or batch number was mentioned	Y- Herbs (1:1:1) were extracted with 70% ethanol using maceration for 3 days with 90 rmp shaking at 40°C, repeated three times. The extract was filtered through a cotton bed followed by Whatman No. 1 filter paper. The filtrate was evaporated under reduced pressure at 45°C using a Buchii Rotaroy Evaporator to leave a gummy concentrate and through vacuum drying.	N	N/A	(121)
BuShenYiQi<br>• *Astragalus mongholicus* Bunge, 1204811<br>• *Epimedium sagittatum* (Siebold & Zucc.) Maxim, 1111018<br>• *Rehmannia glutinosa* (Gaertn.) DC., 1203813<br><br>[Anhui Bencao ChineseMedicine Herb Pieces Co., Ltd (Bozhou, China)]<br><br>No information on the concentration and the ratio of the herbs	Y- Soaked in water for 2h and extracted twice with H_2O at 100°C for 1h, the filtered liquid was concentrated to a clear cream. After spray drying, the spray powder was mixed homogeneously and made into mesh particles.	Y- LC-ESI-Q-TOF-MS	N/A	(68)
Yupingfeng	N	N	N/A	(147)

• *Astragalus mongholicus* Bunge<br>• *Atractylodes macrocephala* Koidz.<br>• *Saposhnikovia divaricata* (Turcz. ex Ledeb.) Schischk.<br><br>[Guangdong HuanQiu Pharmaceutical Company]<br><br>No information on the concentration and ratio of the herbs<br><br>No voucher specimen or batch number was mentioned				
PTQX<br>• *Atractylodes macrocephala* Koidz., 10 g<br>• *Pseudostellaria heterophylla* (Miq.) Pax, 10 g<br>• *Dioscorea oppositifolia* L., 15 g<br>• *Coix lacryma-jobi* var. ma-yuen (Rom.Caill.) Stapf, 20 g<br>• *Imperata cylindrica* (L.) P. Beauv., 15 g<br>• *Forsythia suspensa* (Thunb.) Vahl, 10 g<br>• *Dictamnus dasycarpus* Turcz., 10 g	N	Y- UHPLC-LTQ-Orbitrap-MS	N/A	(139)

• Margarita, 0.3g (no specific species)<br>• *Glycyrrhiza glabra* L., 5 g<br><br>[Jiangyin Tian Jiang Pharmaceutical Co., Ltd. (Jiangsu, China)]<br><br>The formula has been patented for the treatment of AD (patent no.: ZL2013 1 0328668.4, China				
Taeumjowi-tang<br>• *Coix lacryma-jobi* var. ma-yuen (Rom.Caill.) Stapf<br>• *Castanea crenata* Siebold & Zucc.<br>• *Raphanus raphanistrum* subsp. sativus (L.) Domin<br>• *Schisandra chinensis* (Turcz.) Baill.<br>• *Platycodon grandiflorus* (Jacq.) A. DC.<br>• *Acorus gramineus* Aiton<br>• *Ephedra sinica* Stapf<br>• *Liriope muscari* (Decne.) L.H. Bailey<br><br>[I-World Pharm. Co. (Incheon, Republic of Korea)]	N	Y- UPLC	N/A	(107)

No information on the concentration and the ratio of the herbs<br>No voucher specimen or batch number was mentioned				
Jianpi Chushi<br>• *Poria cocos* F.A. Wolf, 20 g<br>• *Dioscorea oppositifolia* L., 20 g<br>• *Dictamnus dasycarpus* Turcz., 20 g<br>• *Zaocys dhumnade*, 15 g (no information in MPNS)<br>• *Atractylodes lancea* (Thunb.) DC., 15 g<br>• *Citrus aurantium* L., 15 g<br>• *Scutellaria baicalensis* Georgi, 10 g<br>• *Sophora flavescens* Aiton, 10 g<br>• *Paeonia lactiflora* Pall., 10 g<br>• *Glycyrrhiza glabra* L., 5 g<br><br>[Tibet Qizheng Tibetan Medicine Ltd.]<br><br>No information on the ratio of the herbs<br><br>No voucher specimen or batch number was mentioned	Y- Boiled for 30 min, filtrated and concentrated.	N	N/A	(133)

Calycosin (pure compound)<br><br>[Tianjin Marker Bio-Tech Co. Ltd. (Tianjin, China)]<br><br>No information on the concentration of the compound	N/A	N/A	≥ 98.75%	(129)
Physcion (pure compound)<br><br>[Cayman Chemical Company (Ann Arbor, MI, USA)]<br><br>No information on the concentration of the compound	N/A	N/A	≥ 98%	(96)
Chrysophanol<br><br>[Sigma Chemical Co.]<br><br>No information on the concentration of the compound	N/A	N/A	N	(37)
Cimifugin (pure compound)<br><br>[National Institutes for Food and Drug Control (Shanghai, China)]<br><br>No information on the concentration of the compound	N/A	N/A	≥ 99%	(135)
Esculetin, 20 mg (112.27μMol)<br><br>[local market (Daegu, Republic of Korea)]	Y- Refluxed twice with 95% ethanol for 2 h. The extracted solution was filtered through a filter paper. The filtrate was concentrated to dryness by a rotary evaporator under reduced pressure below 40°C. The extract was suspended in	Y- NMR	N	(50)

	distilled water and successively partitioned. 5 fractions were re-chromatographed by using silica gel to yield six subfractions.			
Paeonol<br><br>[National Institutes for Food and Drug Control (Beijing, China)]<br><br>No information on the concentration of the compound	N	N	N	(94)
Gintonin<br><br>No information of the supplier and the concentration of the compound	Y- 4-year-old ginseng was ground into small pieces and refluxed with 70% fermented ethanol eight times for 8 h each at 80°C. The extracts were concentrated, dissolved in distilled, cold water and stored at 4°C for 24–96 h. The supernatant and precipitate of water fractionation after ethanol extraction of ginseng was separated by centrifugation. The precipitate after centrifugation was lyophilized.	N	N	(75)
Tryptanthrin<br><br>No information of the supplier and the concentration of the compound	N	N	N	(36)
Thymoquinone<br><br>[Sigma Aldrich (US)]<br><br>No information on the concentration of the compound	N	N	≥ 98%	(5)

N/A: Not applicable or the articles did not mention the parts of the plant used nor the concentration and purity, ESI: electrospray ionization, MS: mass spectrometry, UPLC: ultra-performance liquid

chromatography, HPLC: high-performance liquid chromatography, QTOF: Quadrupole Time-of-Flight, NMR: nuclear magnetic resonance, MPNS: Medicinal Plant Names Services
