# Supplementary material for: Management of Atopic Dermatitis Via Oral and Topical Administration of Herbs in Murine Model: A Systematic Review
Source: Front Pharmacol. 2022 May 24;13:785782. doi: 10.3389/fphar.2022.785782 (PMC9171034; doi:10.3389/fphar.2022.785782)
Supplement: Supplementary file 4 [file Table3.pdf]

Supplementary Table 3: Study parameters and suggested mechanism (s) of the included topical administration of herb and their active constituents' studies

Herb Name	Sample Type	Experimental Outcomes	Proposed mechanism	References
Isodon inflexus (Thunb.) Kudo	Ethanolic extract (Dried whole plant)	↓dermatitis severity<br>↓scratching, ↓histamine<br>↓ear swelling, ↓epidermal thickness, ↓serotonin<br>↓serum IgE, ↓CORT<br>↓IFN-γ, ↓NF-κB, ↓TNF-α<br>↓macrophage activation i.e., CD68 & Iba1<br>↓mast cell count	Attenuation of increase stress levels and depression-related hormones via glucocorticoid-related mechanisms	(112)
Gardenia jasminoides J. Ellis	Ethanolic extract (Dried whole plant)	↓scratching, ↓dermatitis severity<br>↓ear thickness, ↑epidermal thickness<br>↓mast cell count<br>↓IL-4, ↓IL-5, ↓IL-13, ↓TNF-α<br>↑skin barrier proteins (involucrin & loricrin)	Restoration of skin barrier function and suppression of Th2 cytokines<br><br>Analysis on signaling pathway was not performed	(111)
Morus alba L.	Ethanolic extract (Dried whole plant)	↓AD-like symptoms,<br>↓dermatitis severity<br>↓cellular infiltration,<br>↓epidermal hyperplasia<br>↓plasma IgE, ↓plasma histamine	Suppression of TARC production via inhibition of NO and PGE_2 (*in vitro*)<br><br>Analysis on signaling pathway was not performed	(85)
Artemisia capillaris Thunb.	Ethanolic extract (Dried whole plant)	↓AD-like symptoms<br>↓dermatitis severity<br>↓mast cell count<br>↓plasma IgE, ↓plasma histamine	Reduction of histamine by inhibiting IgE-mediated mast cell degranulation<br><br>Analysis on signaling pathway was not performed	(31)
Saxifraga stolonifera (L) Meeb (Saxifragaceae)	Ethanolic extract (Dried whole plant)	↓ear thickness, ↓epidermal thickness<br>↓auricle swelling	Suppression of Th2 cytokines and promotion of Th1 cytokines	(143)

		↓eosinophils, ↓mast cell count,<br>↓IL-4, ↓IL-6<br>↓immune organ weight (thymus & spleen)<br>↑TNF-α & ↑IL-2<br>No significant changes of IFN-γ	Analysis on signaling pathway was not performed	
Spirodela polyrhiza (L.) Schleid.	Ethanolic extract (Leaves)	↓mast cell count<br>↓serum IgE, ↓IL-4, ↓IL-6, ↓TNF-α<br>↑epidermal and dermal hyperplasia<br>↓NF-κB, ↓IκBα, ↓MAP kinase	Suppression of pro-inflammatory mediators via inhibition of MAPK and NF-κB signalling pathways	(77)
Alpinia Intermedia Gagnep	Ethanolic extract (Leaves)	↓AD-like symptoms<br>↓dermatitis severity<br>↓TEWL, ↓scratching<br>↓mast cell degranulation	Abrogation of itch sensation and inhibition of mast cell degranulation	(2)
Pyrus ussuriensis Maxim.	Ethanolic extract (Leaves)	↓TEWL, ↑skin hydration<br>↓erythema, ↓scratching, ↓hypertrophy<br>↓AD-like symptoms, ↓dermatitis severity<br>↓serum IgE,<br>↓cellular infiltration	Suppression of pro-inflammatory cytokines<br><br>Analysis on signalling pathway was not performed	(17)
Pyrus ussuriensis Maxim.	Ethanolic Extract (Leaves)	↓TEWL, ↓AD-like symptoms, ↓scratching, ↓dermatitis severity, ↓epidermal thickness<br>↓serum IgE, ↓IL-1β<br>↓mast cell count, ↓cellular infiltration<br>↑skin barrier proteins (filaggrin & involucrin)	Restoration of skin barrier function & improvement in skin hydration<br><br>Analysis on signalling pathway was not performed	(16)
Urtica thunbergiana Siebold & Zucc.	Ethanolic extract (Leaves)	↓AD-like symptoms, ↓TEWL<br>↓spleen weight, ↓scratching, ↓epidermal thickness<br>↑skin hydration, ↓erythema index, ↑filaggrin<br>↓mast cell count, ↓serum IgE<br>↓MAPKs phosphorylation (p38, ERK), ↑IκBα<br>↓NFATc1 phosphorylation	Suppression of MAPK/NF-κB signalling pathways	(100)

Broussonetia kazinoki Siebold	Ethanolic extract (Leaves)	↓AD-like symptoms, ↓dermatitis score<br>↓dermal thickness, ↓epidermal thickness<br>↓mast cell count<br>↓serum IgE, ↓IL-4	Inhibition of Th2 response via suppression of TARC and MDC binding to CCR4<br><br>Analysis on signaling pathway was not performed	(76)
Wikstroemia indica (L.) C.A. Mey.	Ethanolic extract (Stem & Leaves)	↓AD-like symptoms, ↓epidermal thickness<br>↑skin hydration, ↓TEWL<br>↓mast cell count<br>↓serum IgE, ↓IL-4	Inhibition of IL-4 over expression<br><br>Analysis on signaling pathway was not performed	(83)
Angelica sinensis (Oliv.) Diels	Ethanolic extract (Roots)	↓epidermal &dermal thickness, ↓mast cell count, ↓scratching<br>↓serum IgE, ↓substance P<br>↓IL-4, ↓ IL-6, ↓TNF-α, ↓ IFN-γ<br>↓NF-κB, ↓p- IκBα<br>↓MAPKs phosphorylation (p38, ERK & JNK)	Suppression of Th1 & Th2 cytokines and down-regulation of substance P	(78)
Inula helenium L.	Ethanolic extract (Roots)	↓dermatitis score, ↓right ear swelling<br>↓epidermal thickness, ↓dermal thickness<br>↓cellular infiltration<br>↓serum IgE, ↓TNF-α, ↓IFN-γ<br>↓IL-4, ↓IL-5, ↓IL-13	Down-regulation of Th1/Th2 -associated cytokines<br><br>Analysis on signaling pathway was not performed	(134)
Tribulus terrestris L.	Ethanolic extract (Fruits)	↓AD-like symptoms, ↓TEWL<br>↓dermatitis severity<br>↓eosinophil & ↓mast cell count<br>↓Orai-1 channel	Modulation of calcium ion channel and suppression of mast cell count	(57)
Juniperus rigida Siebold & Zucc.	Ethanolic extract (Fruits)	↓TEWL, ↓AD-like symptoms<br>↓lymphocyte infiltration,<br>↑ skin hydration<br>↓ear thickness,<br>↓epidermal thickness<br>↓serum IgE & IL-4	Restoration of skin barrier function and suppression of serum IgE and IL-4 production<br><br>Analysis on signaling pathway was not performed	(82)

Gardenia jasminoides J. Ellis	Ethanolic extract (Fruits)	↓dermatitis severity, ↓ear thickness<br>↓epidermal thickness, ↓histamine, ↓total IgE<br>↓mast cell count<br>↓serum IL-4, ↓IL-6, ↓TNF- α<br>↓ICAM-1, ↓VCAM-1	Suppression in the production of pro-inflammatory cytokines & expression of adhesion molecules<br><br>Analysis on signalling pathway was not performed	(125)
Forsythia Suspensa (Thunb.) Vahl	Ethanolic extract (Fruits)	↓AD-like symptoms<br>↓dermatitis severity<br>↓ear thickness, ↓epidermal thickness, ↓serum IgE<br>↓mast cell count<br>↓mRNA IL-4, IFN-γ	Suppression of Th2 response, down-regulation of IgE-mediated mast cell activation<br><br>Analysis on signalling pathway was not performed	(124)
Dioscorea quinqueloba Thunb.	Ethanolic extract (Rhizomes)	↓ear swelling, ↓lymphocyte infiltration<br>↓ear thickness, ↓epidermal thickness<br>↓serum IgE, ↓IL-4, ↓TEWL<br>↓skin surface pH, ↓epidermal hyperplasia	Suppression of serum IgE & Th2 cytokine<br><br>Analysis on signalling pathway was not performed	(48)
Coptis chinensis Franch.	Ethanolic extract (Rhizomes)	↓scratching, ↓spleen weight<br>↓AD-like symptoms, ↓dermatitis severity<br>↓skin thickness, ↓serum IgE, ↓IL-4	Inhibition of apoptosis of keratinocytes by reducing key protein expressions (*in vitro*)<br><br>Analysis on signalling pathway was not performed	(137)
Pterocarpus santalinus L.f.	Ethanolic extract (Bark)	↓epidermal thickness, ↓TEWL<br>↓skin hypertrophy, ↓eosinophil<br>↓mast cell count, ↓serum IgE, ↑serum IgG2a<br>↓TNF-α, ↓IL-4, ↓PGE_2	Regulation of serum levels of IgE and IgG2a, along with suppression of Th1 and Th2 cytokines<br><br>Analysis on signaling pathway was not performed	(33)

Stellera chamaejasme L.	Ethanolic extract (Dried aerial parts)	↓AD-like symptoms, ↑skin hydration, ↓TEWL<br>↑lymphocyte infiltration<br>↓ear thickness, ↓epidermal thickness<br>↑mast cell count, ↓mast cell degranulation<br>↓serum IgE, ↓IL-4	Restoration of skin barrier function and inhibition of IL-4 expression<br><br>Analysis on signaling pathway was not performed	(52)
Centella asiatica (L.) Urb.	Ethanolic extract (Dried aerial parts)	↓lymph node weight, ↓AD-like symptoms<br>↓ear thickness, ↓epidermal thickness<br>↓serum IgE, ↓IL-4, ↓IL-13<br>↓TNF-α, ↓IL-1β, ↓COX-2, ↓iNOS, ↓p50/p65 translocation	Suppression of pro-inflammatory mediators via inhibition of NF-κB signaling pathway	(44)
Quercus mongolica subsp. crispula (Blume) Menitsky	Ethanolic extract (Acorn shell)	↓AD-like symptoms, ↑skin hydration, ↓TEWL<br>↓ear thickness, ↓epidermal thickness<br>↓mast cell count, ↓eosinophil infiltration<br>↓serum IgE, ↓IL-4<br>↓ mRNA IL-25, ↓IL-33, ↓TNF-α, ↓IL-1β	Down-regulation of serum IgE levels and inhibition of mRNA gene expression of Th2-type cytokines<br><br>Analysis on signalling pathway was not performed	(81)
Cucumis melo L.	Ethanolic extract (Fruit pulp)	↓AD-like symptoms, ↓dermatitis severity<br>↓epidermal hyperplasia<br>↓mast cell count<br>↓plasma IgE, ↓plasma histamine	Suppression of IgE<br><br>Detailed analysis on signalling pathway was not performed	(32)
Panax ginseng C.A. Mey.	Methanolic Extract (N/A)	↓P-p70S6K<br>↓p-mTOR	Inhibition of p70S6K phosphorylation in infiltrating basophils	(104)
Acer tegmentosum Maxim.	Methanolic extract (Bark)	↓dermatitis severity<br>↓dermal thickness, ↓epidermal thickness<br>↓dermal mast cells count, ↓IL-4, ↓IL-5, ↓IL-13<br>↓serum IgE, ↓TARC<br>↓TSLP (mRNA expression & production)	Restoration of Th1/Th2 balance by reducing TSLP production<br><br>Analysis on signaling pathway was not performed	(140)
Cyperus rotundus L.	Methanolic extract (Rhizomes)	↓dermatitis severity, ↓epidermal thickness<br>↓cellular infiltration, ↓hyperkeratosis	Restoration of skin barrier function, inhibition of IgE and	(142)

		↓scratching, ↑filaggrin,<br>↓Serum IgE<br>↓IL-6, ↓IL-1β, ↓IL-13	pro-inflammatory cytokine production<br><br>Analysis on signaling pathway was not performed	
Persicaria tinctoria (Aiton) Spach	Distilled water extract (Dried whole plant)	↓AD-like symptoms,<br>↓epidermal thickness<br>↓scratching<br>↓cellular infiltration<br>↓TSLP, ↓histamine<br>↓serum IgE, ↓IL-4, ↓IL-6	Blockade of caspase-1/receptor-interacting protein 2 pathway in stimulated mast cells	(38)
Vincetoxicum atratum (Bunge) C. Morren & Decne.	Distilled water extract (Roots)	↓scratching<br>↓epidermal & dermal thickness,<br>↓serum IgE, ↓skin mast cell<br>↓IL-4, ↓ IL-6, ↓TNF-α, ↓IL-1β<br>↓NF-κB, ↓ IκBα, ↓MAPK	Suppression of pro-inflammatory mediators and Th2 response via inhibition MAPK and NF-κB signaling pathways	(23)
Pseudostellaria heterophylla (Miq.) Pax	Distilled water extract (Roots)	↓dermal thickness, ↓epidermal thickness<br>↓mast cell count, ↓serum IgE,<br>↓$CD4^+$ T-cell recruitment<br>↓IL-4, ↓IL-6, ↓IL-8<br>↓IL-1β, ↓TNF-α, ↓IFN-γ<br>↓p- IκBα, ↓p-JNK, ↓p-ERK1/2,<br>↓p-p38	Suppression of MAPK/NF-κB signaling pathways	(21)
Rosa multiflora Thunb.	Acetone extract (Roots)	↓AD-like symptoms<br>↓dermatitis severity<br>↓iNOS, ↓COX-2,<br>↓eosinophil ratio<br>↓serum IgE, ↑IL-10<br>↓IL-4, ↓IL-5, ↓IL-13	Suppression of Th2 response via activation of Treg<br><br>Analysis on signaling pathway was not performed	(110)
C2RLP<br>• *Rosa multiflora* Thunb.<br>• *Lespedeza bicolor* Turcz.<br>• *Platycladus orientalis* (L.) Franco	Mixture (Ethanolic extracts)	↓dermatitis severity,<br>↓cellular infiltration<br>↓epidermal hyperplasia<br>↓IL-4, ↓nitrite (NO), ↓PGE2,<br>↓TARC	Suppression of Th2 cytokines and pro-inflammatory mediators	(92)

• *Castanea crenata* Siebold & Zucc.<br>• *Cornus officinalis* Siebold & Zucc.				
Dangguibohyul-Tang<br>• *Astragalus mongholicus* Bunge<br>• *Angelica sinensis* (Oliv.) Diels	Mixture (Ethanolic extracts)	↓AD-like symptoms, ↓scratching<br>↓dermal thickness, ↓epidermal thickness<br>↓mast cell count, ↓serum IgE<br>↓IL-4, ↓IL-6, ↓IL-1β, ↓TNF-α, ↓IFN-γ<br>↓NF-κB, ↓p- IκBα<br>↓MAPKs phosphorylation (p38, ERK & JNK)	Suppression of both Th1 & Th2 cytokine production via the inhibition of MAPK and NF-κB signalling pathways	(22)
Danggui Buxue Tang<br>• *Astragalus mongholicus* Bunge<br>• *Angelica sinensis* (Oliv.) Diels	Mixture (Ethanolic extracts)	↓AD-like symptoms, ↓ear swelling<br>↓epidermal thickness, ↓eosinophil infiltration<br>↓mast cell count, ↓serum IgE, ↓IgG1<br>↓IL-4, ↓TNF-α	Suppression of Th1 &Th2 cytokines<br><br>Analysis on signalling pathway was not performed	(28)
KAJD<br>• *Phellodendron amurense* Rupr.<br>• *Sesamum indicum* L.<br>• *Sophora flavescens* Aiton<br>• *Glycyrrhiza glabra* L.<br>• *Ophiopogon japonicus* (Thunb.) Ker Gawl.<br>• *Radix rehmanniae* Exsiccat	Mixture (Water extracts)	↓AD-like symptoms,<br>↓$CD4^+$ T-cell recruitment<br>↓mast cell count, ↓eosinophil count<br>↓serum IgE<br>↓IL-6, ↓IL-10, ↓IL-12	Suppression of Th2 response by controlling transcriptional expression of cytokines<br><br>Analysis on signalling pathway was not performed	(42)

Pentaherbs<br>• *Lonicera japonica* Thunb.<br>• *Mentha canadensis* L.<br>• *Paeonia* × *suffruticosa* Andrews<br>• *Atractylodes lancea* (Thunb.) DC.<br>• *Phellodendron amurense* Rupr.	Mixture (Water extracts)	↓ear redness, ↓ear swelling (only ORAL)<br>↓epidermal thickness, ↓eosinophil infiltration<br>↓mast cell count<br>↓serum IL-5, ↓IL-12	Inhibition of pro-inflammatory cytokine production, IL-12 & eosinophil activator, IL-5<br><br>Analysis on signalling pathway was not performed	(131)
Huang-Lian-Jie-Du-Tang (HLJDT)<br>• Coptis chinensis Franch<br>• Scutellaria baicalensis Georgi<br>• *Phellodendron amurense* Rupr.<br>• *Gardenia jasminoides* J Ellis	Mixture (Water extracts)	↓AD-like symptoms, ↓cellular infiltration<br>↓ear swelling, ↓hyperkeratosis<br>↓IL-2, ↓IL-4, ↓IL-5, ↓IL-6<br>↓IL-1α, ↓IL-1β, ↓NF-κB<br>↓IκB-α, ↓IFN-γ, ↓TNF-α<br>↓$CD4^+/CD8^+$ T-lymphocyte ratio<br>↓MAPKs phosphorylation (p38, ERK & JNK)	Suppression of pro-inflammatory mediators via inhibition of MAPKs, IκB-α & NF-κB signalling pathways	(27)
Si-Ni-San<br>• *Bupleurum chinense* DC.<br>• *Paeonia lactiflora* Pall.<br>• *Glycyrrhiza uralensis* Fisch. ex DC.<br>• *Citrus aurantium* L.	Mixture (Water extracts)	↓AD-like symptoms, ↓cellular infiltration<br>↓IL-2, ↓IL-4, ↓IL-6<br>↓IL-1α, ↓IL-1β, ↓TNF-α<br>↓$CD4^+/CD8^+$ T-lymphocyte ratio, ↓epidermal hyperplasia, ↓IκB-α, ↓NF-κB<br>↓MAPKs phosphorylation (p38, ERK & JNK)	Suppression of MAPKs signalling pathway activation	(26)
Atofreellage		↓dermal thickness, ↓epidermal thickness	Regulation of IgE and histamine production	(67)

• *Rhus javanica* Linne<br>• *Kochi scoparia* Schrader<br>• *Cnidium monnieri* (L.) Cusson<br>• Houttuynia cordata Thunb.<br>• *Nepeta tenuifolia* Benth.<br>• *Sophora flavescens* Aiton<br>• *Rheum palmatum* L.<br>• *Lithospermum erythrorhizon* Siebold & Zucc.<br>• *Terminalia chebula* Retz.<br>• *Trichosanthes kirilowii* Maxim.	Mixture (Distilled water extracts)	↓mast cell count, ↓leukocyte count<br>↓serum IgE, ↓serum histamine<br>↓IL-4, ↓IL-5, ↓IL-13<br>↓IL-6, ↓IL-1β, ↓TNF-α, ↓NF-κB<br>↓MAPKs phosphorylation (p38, ERK & JNK)	by inhibition of Th2 response via MAPK and NF-κB signalling pathways	
• *Aucklandia costus* Falc.<br>• Platycladus orientalis (L.) Franco	Mixture (1,3-butylene glycol extracts)	↓AD-like symptoms<br>↓dermis thickness<br>↓epidermal thickness<br>↓eosinophil,<br>↓mast cell count<br>↓serum IgE, ↓IL-4, ↓IL-13<br>↓ mRNA TNF- α & ↓IFN- γ	SC: Suppression of IFN- γ & TNF- α activation by inhibiting STAT1 phosphorylation<br><br>TOL: Suppression of pro-inflammatory cytokines mainly TNF- α and IL-1β<br><br>Detailed analysis on signalling pathway was not performed	(141)

Jawoongo<br>• *Arnebia euchroma* (Royle ex Benth.) I.M. Johnst.<br>• *Angelica gigas* Nakai	Mixture (N/A)	↓skin thickness, ↓WBCs<br>↓mast cell count<br>↓serum IgE, ↓IL-6, ↓IL-10, ↓IL-12, ↓IL-2, ↓IL-4, ↓IL-13, ↓TNF-α	Down-regulation of serum IgE levels and suppression of Th1 & Th2 cytokines<br><br>Analysis on signalling pathway was not performed	(70)
Stewartia pseudocamellia Maxim.	5,6-dihydroergosteol-glucoside (isolated compound)	↓lymph node weight, ↓spleen weight<br>↓AD-like symptoms, ↓epidermal thickness<br>↓eosinophil, ↓mast cell count<br>↓CCL17, ↓CCL22<br>↓histamine, ↓total IgE	Suppression in the production of Th2 chemokines<br><br>Analysis on signalling pathway was not performed	(55)
Ophiopogon japonicus (Thunb.) Ker Gawl.	Ophiopogonin D (isolated compound	↓epidermal thickness, ↓spleen weight<br>↓IL-4, ↓IL-5, ↓IL-13, ↓eotaxin<br>↓TNF-α, ↓IFN-γ, ↓IL-1β mRNA<br>↓mast cell count	Suppression of mast cell activation and inhibition of mRNA gene expression of Th2-type cytokines<br><br>Analysis on signalling pathway was not performed	(4)
Fritillaria usuriensis Maxim.	Peiminine (isolated compound)	↓epidermal thickness, ↓dermal thickness<br>↓eosinophil, ↓mast cell count<br>↓serum IgE, ↓IL-4, ↓IL-6, ↓IL-13<br>↓NF-κB, ↓TNF-α<br>↓MAPKs phosphorylation (p38, ERK & JNK)	Suppression of pro-inflammatory mediators via inhibition of NF-κB & MAPK signalling pathways	(88)
Artemisia scoparia Waldst. et Kitaib	3,5-dicaffeoyl-epi-quinic acid (isolated compound)	↓AD-like symptoms, ↓scratching<br>↓epidermal thickness<br>↓mast cell count, ↓eosinophil infiltration<br>↓serum histamine, ↓serum IgE<br>↓TNF- α, ↓caspase-1 activation	Regulation of caspase-1 signalling pathways	(115)
Blainvillea acmella (L.) Philipson	Spilanthol (isolated compound)	↓ear thickness, ↓collagen deposition<br>↓mast cell count, ↓eosinophils<br>↓serum IgE, ↓IgG1, ↑IgG2a<br>↓MAPK proteins, ↓COX-2, ↓iNOS	Restoration of Th1/Th2 balance, inhibition of MAPK pathway and mast cell infiltration	(46)

Soy isoflavone daidzin	*7,8,4'-Trihydroxyisoflavone* (isolated compound)	↓AD-like symptoms, ↓dermatitis severity, ↓TEWL ↓ear thickness, ↓epidermal hypertrophy, ↓scratching ↓serum IgE, ↓eosinophil, ↓mast cell count, ↓TARC ↓IL-4, ↓IL-5, ↓IL-13 ↓IL-12, ↓IFN-γ	Suppression of Th1 & Th2 cytokines<br>Analysis on signalling pathway was not performed	(63)
Gardenia jasminoides J. Ellis	Crocin (isolated compound)	↓dermatitis severity, ↓eosinophil ↓mast cell count ↓ear thickness, ↓epidermal thickness, ↓serum IgE ↓IL-4, ↓IL-5, ↓IL-13 ↓TARC, ↓p-STAT6, ↓IκBα	Suppression of Th2 response via inhibition of NF-κB /STAT6 signalling pathways	(123)
Stellera chamaejasme L.	Stechamone (isolated compound)	↓stratum corneum thickness ↓dermal thickness, ↓lymphocyte infiltration ↓mast cell count ↓mast cell densities, ↓TEWL ↓serum IgE, ↓IL-4	Inhibition of IL-4 expression, regulation of IgE synthesis and suppression of mast cell degranulation<br>Analysis on signalling pathway was not performed	(52)
Alpinia officinarum Hance	Galangin (isolated compound)	↓dermal thickness, ↓epidermal thickness ↓eosinophil, ↓mast cell count ↓total IgE, ↓DfE-specific IgE, ↓IgG2a ↓IL-4, ↓IL-5, ↓IL-13 ↓IL-31, ↓IL-32, ↓IFN- γ	Suppression of Th1 & Th2 cytokines, with IL-32 as its target<br>Analysis on signalling pathway was not performed	(19)
Artemisia species (Species was not mentioned)	Eupatilin (isolated compound)	↓AD-like symptoms, ↓ear thickness, ↓skin barrier protein expression (filaggrin & loricrin) ↓serum IgE, ↓IL-4, ↑PPAR-α ↓ mRNA TNF- α, ↓IFN- γ, TSLP ↓mRNA IL-25, ↓IL-33, ↓IL-1β	Suppression of Th2 cytokines & IL-33 mRNA expression<br>Suppression of TARC inflammation via the inhibition of TSLP cytokine expression	(56)

			Analysis on signalling pathway was not performed	
Origin of the herb was not mentioned	Quercetin (compound)	↓epidermal thickness, ↓hyperplasia<br>↓IL-4, ↓IL-6, ↓CCL17, ↓CCL22<br>↓IFN-γ, ↓TNF-α<br>↓mast cell count	Suppression of Th1 & Th2 cytokines via TLR2 and TLR6 signalling pathway	(45)
Nigella sativa L.	Thymoquinone (isolated oil)	↓ear clinical score (dermatitis severity)<br>↓ear thickness & weight<br>↓total leukocyte count<br>↓serum IgE<br>↓mRNA IL-4, IL-5 & IFN-γ	Suppression of both Th1 & Th2 cytokines<br><br>Analysis on signalling pathway was not performed	(5)
Labiatae or mint family	(R)- (+)- Pulegone (isolated oil)	↓AD-like symptoms, ↓scratching<br>↓dermal thickness, ↓epidermal thickness<br>↓mast cell count, ↓total IgE, ↓nerve growth factor<br>↓IL-4, ↓IL-6, ↓IL-1β, ↓TNF-α, ↓IFN-γ<br>↓MAPKs phosphorylation (p38, ERK & JNK)<br>↓p- IκBα/ IκBα ratio	Suppression MAPK/NF-κB signalling pathways and cytokine production	(24)

N/A: Part of the plant or type of extract was not mentioned
